# Early detection of colorectal cancer somatic mutations using cfDNA liquid biopsies in a murine carcinogenesis model

**DOI:** 10.7150/jca.76516

**Published:** 2022-09-25

**Authors:** Héctor Martínez-Gregorio, Clara Estela Díaz-Velásquez, Mario Esaú Romero-Piña, Miguel Ruiz De La Cruz, Norma Laura Delgado-Buenrostro, Aldo De La Cruz-Montoya, Yolanda Irasema Chirino, Luis Ignacio Terrazas, Luis Alberto Medina, Felipe Vaca-Paniagua

**Affiliations:** 1 Laboratorio Nacional en Salud, Diagnóstico Molecular y Efecto Ambiental en Enfermedades Crónico-Degenerativas, Facultad de Estudios Superiores Iztacala, Tlalnepantla 54090, México.; 2 Unidad de Biomedicina, Facultad de Estudios Superiores Iztacala, UNAM, Tlalnepantla 54090, México.; 3 Unidad de Investigación Biomédica en Cáncer INCan-UNAM, Instituto Nacional de Cancerología, Ciudad de México 14080, México.; 4 Avenida Instituto Politécnico Nacional # 2508, Colonia San Pedro Zacatenco, Delegación Gustavo A. Madero, C.P. Departamento de Infectómica y Patogénesis Molecular, Centro de Investigación y de Estudios Avanzados del Instituto Politécnico Nacional (CINVESTAV-IPN), Mexico City 07360, Mexico; 5 Instituto de Física, Universidad Nacional Autónoma de México, Ciudad de México 04510, México.; 6 Subdirección de Investigación Básica, Instituto Nacional de Cancerología, Ciudad de México 14080, México.

**Keywords:** AOM/DSS model, cfDNA, liquid biopsy, early detection, biomarkers, colorectal cancer, polyclonality.

## Abstract

Colorectal cancer (CRC) is one of the top five cancers in incidence and mortality worldwide. The early detection of this neoplasm through analysis of circulating free DNA (cfDNA), which carries tumor genetic alterations, as a liquid biopsy, could have a major impact in enhancing early detection and reducing the mortality rate. The aim of this work was to demonstrate the feasibility of using cfDNA as a liquid biopsy for the early detection of CRC. For this purpose, we implemented an azoxymethane and dextran sodium sulfate-induced murine carcinogenesis model to detect oncogenic somatic mutations in *Ctnnb1* and *Kras* during CRC development. To enhance the sensitivity in the detection, E-ice-COLD-PCR was utilized to selectively enrich for mutant alleles, followed by massively parallel sequencing. Driving somatic mutations were detected in *Ctnnb1* and *Kras* in the liquid biopsies of early stages of tumor development, corresponding to the formation of aberrant crypt foci, the first histological alterations that can be identified throughout the formation of CRC. The concentration of cfDNA was increased along the carcinogenic process. Polyclonality in *Ctnnb1* was found in tumor samples and cfDNA in this model. On the other hand, the use of cfDNA as a non-invasive test resulted in superior early detection compared to microPET/CT imaging. As a proof-of-principle, this study shows the great potential use of allelic-specific PCR for the detection and enrichment of pathogenic alleles present in cfDNA samples, as a test for early non-invasive detection of CRC. This work provides scientific evidence to set methodological bases that allow early detection of mutations in cfDNA obtained from plasma of CRC in humans.

## Introduction

Colorectal cancer (CRC) is among the top five cancers with higher incidence and mortality worldwide 1. The evolution of CRC from aberrant crypt foci (ACF) to carcinoma *in situ* can take decades and it is asymptomatic, making early detection and therapeutic success challenging. Detection of CRC relies on colonoscopy, histopathological analysis, imaging, and serum tumor marker evaluation, however, these strategies still do not fully satisfy clinical needs due to their lack of sensitivity and specificity to detect early colorectal tumors 2-4.

In the clinic, the use of tumor biopsies is the gold standard for the diagnosis and molecular analysis. However, most patients are biopsied in locally advanced stages and tissue biopsies might not reflect intratumor heterogeneity present in the tumor. On the other hand, the use of liquid biopsies, such as cfDNA in blood plasma, is a recent approach that might allow early detection and capture a more comprehensive molecular profile of CRC in a minimally invasive manner. The use of cfDNA for the early detection of CRC has been suggested, however, these malignancies are often detected in advanced stages which limits their use. For this reason, several reports describe the potential use of cfDNA in CRC management as a marker of tumor postoperative recurrence 5-7, and to assess the tumor burden in metastatic patients 8,9 due to the general lack of access to human samples in the earliest stages of CRC development.

Consequently, the aim of this work was the identification of somatic mutations associated with early stages of CRC development using cfDNA in a well-established *in vivo* model of induced carcinogenesis. First, we set the *in vivo* model of CRC to recapitulate the entire biological process that occurs during CRC development. Second, we implemented an allele-specific PCR methodology to selectively enrich and identify known mutant alleles associated with early stage of CRC development, together with massive parallel sequencing of cfDNA. Third, the use of microPET/CT imaging allowed us to evaluate the development of the tumor and have a closer view of the clinical scenario. With these strategies, we were able to detect driving somatic mutations in cfDNA in aberrant crypt foci, the first histological alterations that can be identified throughout the formation of CRC. The use of cfDNA as a non-invasive test resulted in superior early detection compared to microPET/CT imaging. In addition, the polyclonality of *Ctnnb1* was an interesting finding not previously observed in this model.

As a proof-of-principle, the results of this work provide evidence in the direction of establishing methodological bases that allow the early detection of somatic alterations in plasma associated with CRC in humans.

## Materials and methods

### CRC induction

**Figure 1:**
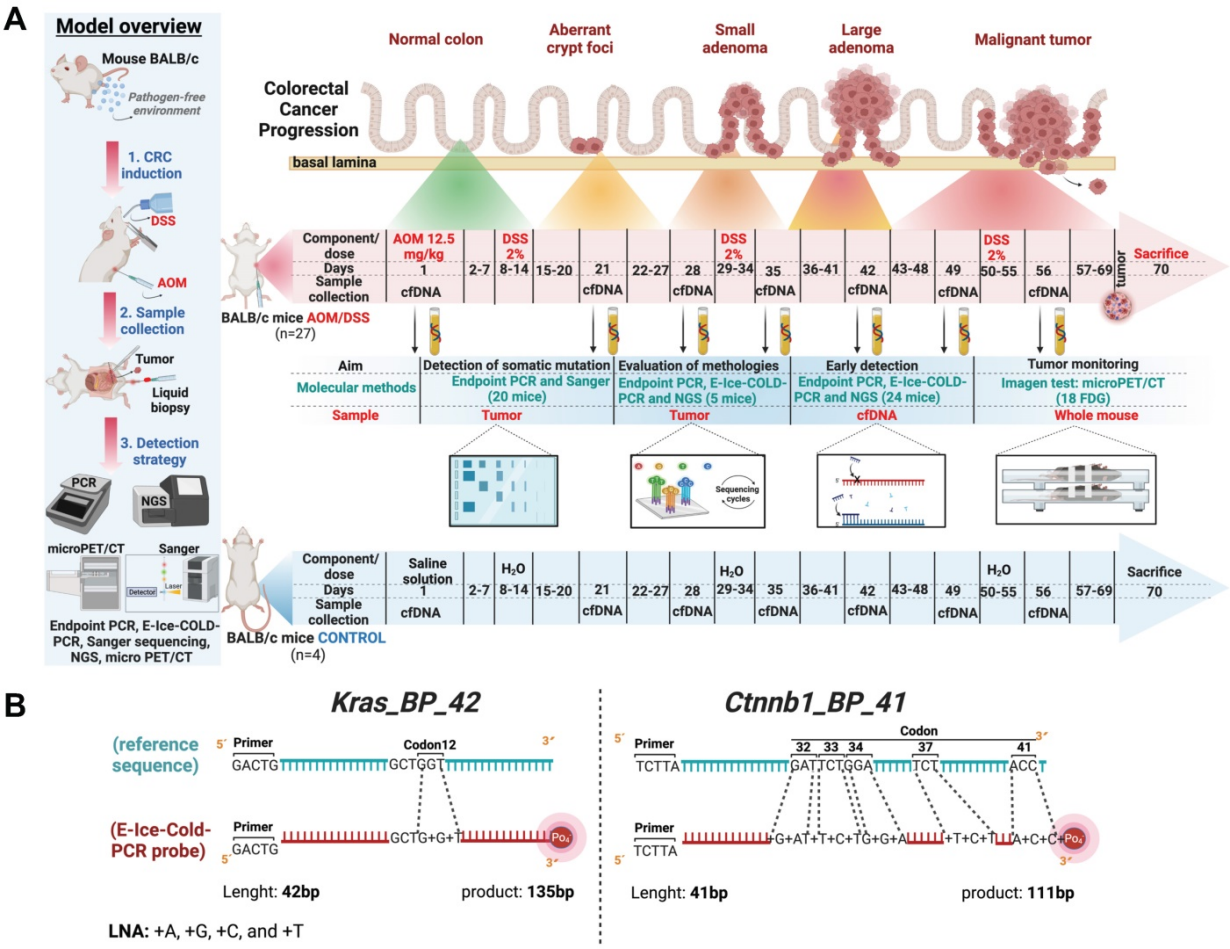
**Experimental design and the azoxymethane and dextran sodium sulfate-induced (AOM/DSS) murine carcinogenesis model for the detection of somatic driving mutations in early stages and blocker probes used in E-ice-COLD-PCR to enrich mutant alleles. (A)** The image depicts the induction of CRC, as well as the molecular and imaging tests used in this study. Blood sampling and microPET/CT were set according to cancer development in this AOM/DSS model. **(B)** Blocker probes used to selectively enrich mutant alleles in *Ctnnb1* and *Kras*.

In this study, we used 31 male BALB/c mice, five- to six-week-old, purchased from Harlan Laboratories (Mexico) and maintained in a pathogen-free environment at the Facultad de Estudios Superiores Iztacala (FES-I), Universidad Nacional Autónoma de México (UNAM) animal facilities. Mice were housed in plastic cages with food and water *ad libitum* and distributed into 2 groups: control group (4 mice) and AOM/DSS group (27 mice). Mice from the AOM/DSS group received an intraperitoneal (i.p.) injection of azoxymethane (AOM) (Sigma-Aldrich Cat. A5486) dosed at 12.5 mg/kg of body weight on day 1; the control group got an i.p. injection of saline solution. Seven days later, 2% dextran sulfate sodium (DSS) (MP Biomedicals Cat. 160110) was supplied in drinking water *ad libitum* for 7 days, followed by water alone for the next 14 days, to repeat two additional DSS cycles in the AOM/DSS group (Figure 1A) 10,11. The entire protocol lasted 70 days and at the end of the experiment, mice were sacrificed, and colorectal tumors were excised and stored at -80°C. All experimental procedures were in strict accordance with the recommendations in the Guide for the Care and Use of Laboratory Animals of the National Institutes of Health (USA) and this protocol was approved by the Committee on the Ethics of Animal Experiments of Facultad de Estudios Superiores Iztacala (CE/FESI/042017/1168).

### Extraction and quantification of DNA

Blood samples were collected during days 1, 21, 28, 35, 42, 49, 56 and 70 for both groups. Blood samples were taken through an incision in the tail of the mice and collected by dripping in a microtainer tube with EDTA (BD Microtainer Cat. 365974). cfDNA extraction was performed with 63 - 200 μL of plasma with the Plasma/Serum Cell-Free Circulating DNA Purification Mini kit (Norgen Biotek Corp, Cat. 55100) according to the manufacturer's recommendations. DNA from Tumor samples were extracted with the DNeasy Blood & Tissue Kit (Qiagen, Hilden, Germany), following the manufacturer's instructions. DNA extracted from both biological resources was quantified by fluorometry (Qubit, Life Technologies) and stored at -20°C until use.

### Tumor metabolic activity and imaging analysis

Tumor follow-up was performed with microPET/CT using 2-[18F]-fluoro-2-deoxy-D-glucose (18FDG), which evaluates the glycolytic activity of tumor cells, that is greater than in normal cells, 12,13 on days 1, 21, 28, 35, 42, 49 and 70. Twelve hours before taking microPET/CT, mice received food with contrast medium iodixanol 270 (Visipaque) and water *ad libitum*. Then, 7.4 - 9.25 MBq (200-250 μCi) of 18FDG were administered intravenously to the AOMDSS group. After 20 min of 18FDG biodistribution, mice were placed in the microPET/CT scan under inhaled anesthesia (oxygen at 800 mmHg and isoflurane 1.5%). MicroPET/CT scans were performed in an Albira ARS microPET/SPECT/CT equipment (Bruker, Spain) using a full-body emission protocol with 10 min for PET and 600 projections (tube voltage 35 kV and 0.2 mA) for CT scans. PET images were reconstructed with Albira's reconstruction software based on the ordered subsets expectation-maximization (OSEM) routine (three iterations). The total lesion glycolysis (TLG) value was calculated from the images using PMOD software (PMOD-Technologies LLC, Zurich), a quantitative value that measures the concentration of 18FDG present in a metabolically active tissue (i.e., the standard uptake value: SUV). TLG is helpful to compare the SUV between different tumors or in the same tumor at different times.

### Primer design and block probes

We designed two sets of primers compatible with E-ice-COLD-PCR (Enhanced-Improved and Complete Enrichment CO-amplification at Lower Denaturation temperature Polymerase Chain Reaction) and endpoint PCR to amplify the mutated regions of mice *Ctnnb1* exon 3, codon 32, 33, 34, 37, and 41; and *Kras* exon 2, codon 12 (Table S1).

Two blocker probes (BPs) for *Ctnnb1* and *Kras* were designed and used in E-ice-COLD-PCR for the enrichment of mutant alleles in the model of AOM/DSS, as described elsewhere 14,15; Figure 1B and Table S2. The BPs were directed towards the wild-type alleles, with a 5-base overlap with the forward primers of *Ctnnb1* and *Kras*, and the 3' end had a phosphorylated group. To maximize the Tm between the wild-type and mutant allele, BPs contained locked nucleic acids (LNAs) at the site where we expect to find the mutations, causing the mutant allele to denature preferentially at a lower temperature than wild type allele.

### Endpoint PCR and E-ice-COLD-PCR

We amplified *Ctnnb1* and *Kras* in the tumor samples and the cfDNA using E-ice-COLD-PCR and endpoint PCR. E-ice-COLD-PCR used a critical temperature (T_c_), defined as the temperature at which the mutant allele that is linked to the BP is preferentially denatured, because it hybridizes imperfectly with the probes and its melting temperature (Tm) is lower than wild-type allele. T_c_ was defined by high resolution melting analysis (HRMA). For the determination of the T_c_ of *Ctnnb1* and *Kras*, three different templates were used: wild-type allele, mutant allele, and an equimolar mixture of both, which were amplified by endpoint PCR using 1 ng of these molecules with the HRM Fast PCR kit (KAPA cat. KK420). The products obtained from PCR were quantified and mixed with the BP at a molarity of 1:1 (PCR product: BP), and analyzed by HRMA (0.3 °C per acquisition, 2 sec hold before each acquisition), from 75-90°C. Once the T_c_ was determined for *Ctnnb1* and* Kras*, we used cfDNA to perform the E-ice-COLD-PCR with 50 nm of the BP based on previous studies 14,15.

### Sanger sequencing and massively parallel sequencing

To detect mutations in the tumor samples, we performed Sanger sequencing in the Applied Biosystems 3500xL Genetic Analyzer. Libraries were prepared at the Laboratorio Nacional en Salud: Diagnóstico Molecular y Efecto Ambiental en Enfermedades Crónico-Degenerativas, FESI, UNAM (Figure S1) to detect mutations in the tumor samples and cfDNA, and sequenced in the MiSeq platform (Illumina). Fastq files were aligned with the mm10 mouse reference genome with the galaxy bioinformatics platform (https://usegalaxy.org) using bwa-mem 16. Somatic mutations were detected with Mutect2 17 and visualized in IGV 18.

## Statistical analysis

The evaluation of significant differences was performed by a one-way analysis of variance (ANOVA), followed by Tukey's multiple comparison tests or by unpaired two-tailed t-tests with GraphPad Prism 5 (San Diego, CA, USA). A linear relationship between cfDNA concentration and days was evaluated by scatterplot and linear regression analysis with correlation coefficient R.

## Results

### Tumor development in the AOM/DSS model

**Figure 2:**
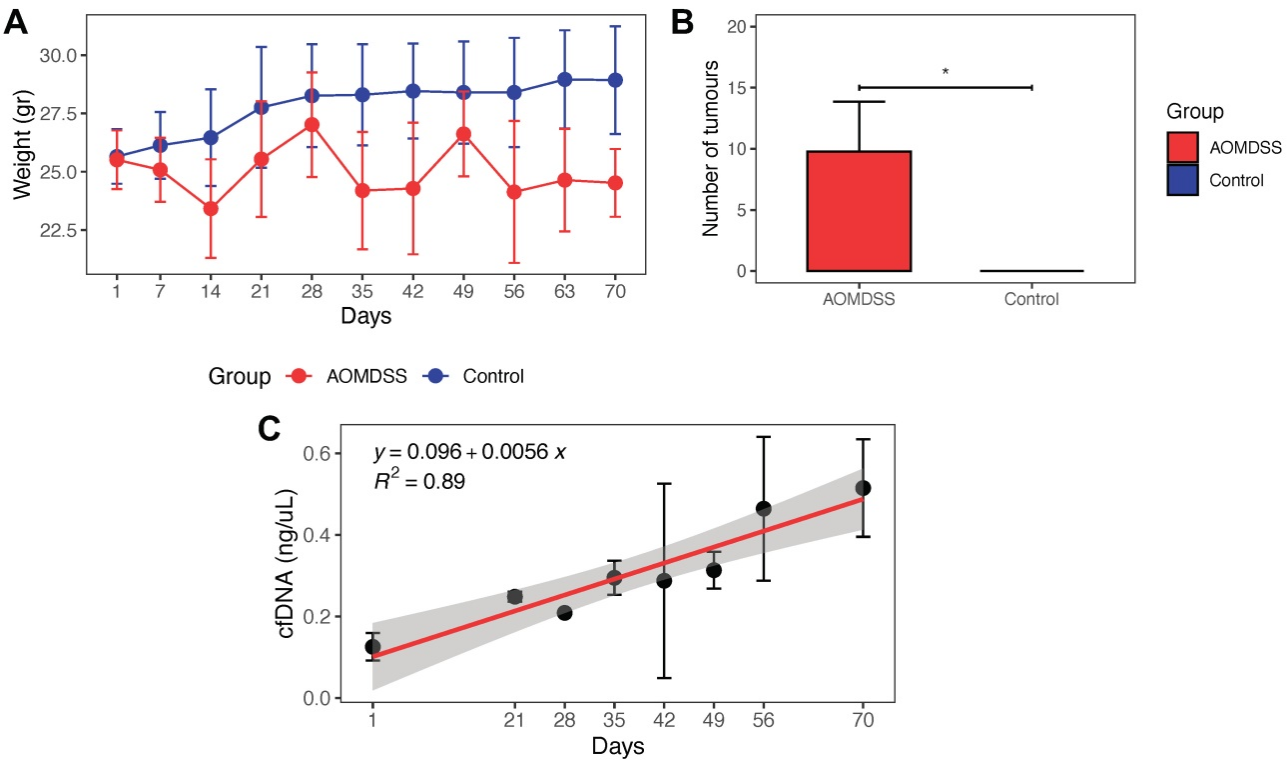
** Physiological changes and tumor development in AOM/DSS model. (A)** Weight of the mice of the AOM/DSS and control groups during the CRC development. **(B)** Number of tumors developed in the AOM/DSS group (n = 22 mice) and control group (n = 4 mice). **(C)** Concentration of cfDNA during tumor progression. The concentration of cfDNA was measured in triplicate. Correlation between cfDNA concentration and days was evaluated by linear regression analysis. Differences in the number of tumors in AOM/DSS group and control group are presented as mean ± SD analyzed by unpaired two-tailed t-tests (*p < 0.05).

To carry out an improved detection in early driving mutations in CRC, we implemented the AOM/DSS model in our laboratory (Figure 1A). During the carcinogenic process, the AOM/DSS group exhibited piloerection, diarrhea, bleeding, and anal protrusion, as well as loss of body weight after each cycle of DSS compared to the control group (Figure 2A).

At the end of the treatment (day 70), mice were sacrificed for macroscopic evaluation of the colon and tumor excision. The AOM/DSS group developed middle and distal colorectal tumors, with an average of 9.8 ± 4.1 tumors per mouse, (Figure 2B, S2). Throughout the duration of the treatment, plasma cfDNA was extracted from 3 mice from the AOM/DSS group at each sampling. An increase in plasma cfDNA concentration was observed in this group during the carcinogenic process (Figure 2C).

### Detection of microadenomas with microPET/CT

**Figure 3:**
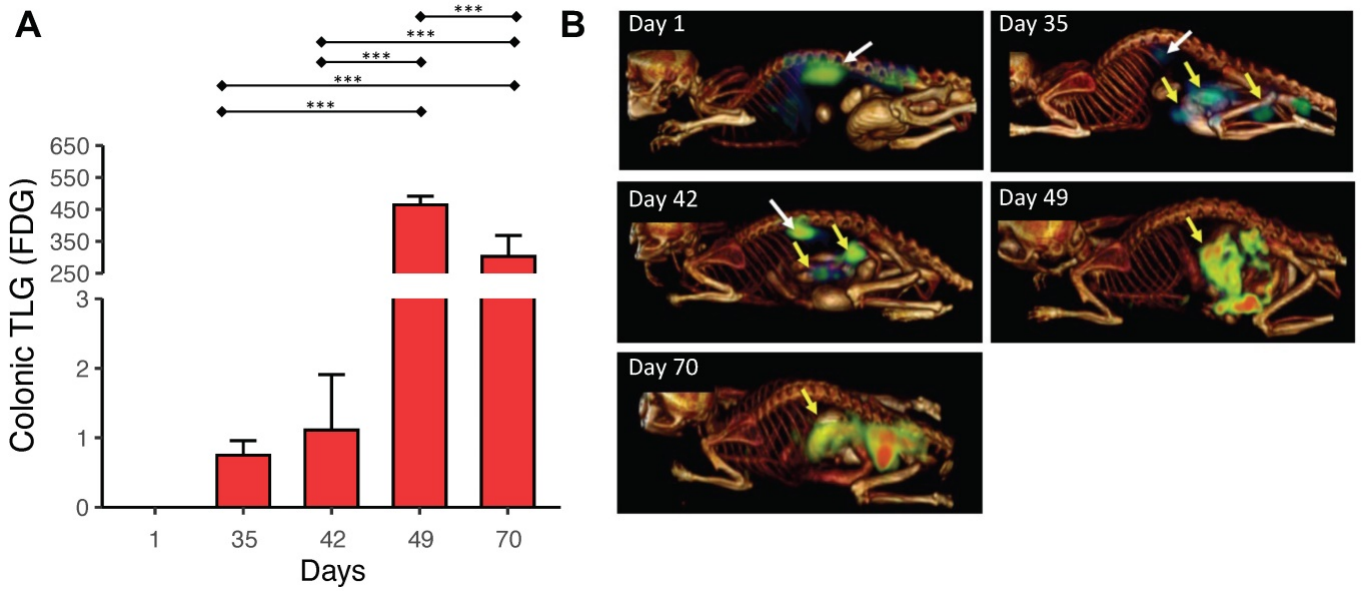
** Detection of microadenomas with microPET/CT imaging. (A)** Total lesion glycolysis (TLG) values on specific days of tumor growth calculated from the images. **(B)** Representative microPET/CT images from the same animal on specific days. Yellow and white arrows show 18FDG uptake in the colon and the brown fat, respectively. MicroPET/CT taking was performed in triplicate. Data are presented as the mean ± SEM analyzed by a one-way ANOVA followed by Tukey's multiple comparison test (*** p < 0.001).

Tumor growth during the carcinogenic process was followed with microPET/CT imaging using 18FDG as a molecular probe. Tumor metabolic activity was determined in terms of the Total Lesion Glycolysis (TLG), in units of standard uptake value (SUV), in the colon on day 36 in the AOM/DSS group, a stage that corresponds to the formation of microadenomas in this model (Figure 3A). The microPET/CT images of the same mouse at different times (Figure 3B) illustrate the tumor growth in the colon.

### Colorectal tumors exhibit polyclonality in *Ctnnb1*

**Figure 4:**
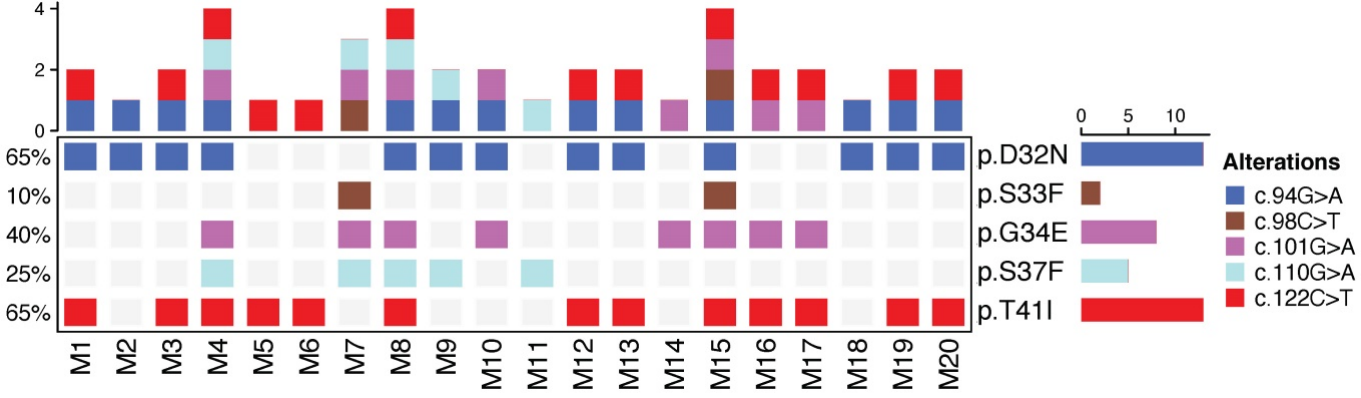
** Allelic distribution of *Ctnnb1* in tumor samples.** Oncoprint depicts mutations found in *Ctnnb1* in 20 tumor samples analyzed from 20 mice of the AOM/DSS group in the day 70 that corresponds to the development of colorectal cancer in this model. Color codes show different alleles mutated in *Ctnnb1*. Alterations are shown at the protein and cDNA level.

To analyze the tumor genotype in *Ctnnb1* and *Kras*, endpoint PCR and Sanger sequencing were done in 20 tumor samples from 20 mice of the AOM/DSS group. Mutations in *Ctnnb1* were detected in all samples at codons 32, 33, 34, 37 and 41; in *Kras* were not detected (Figure 4). In addition, more than two different mutations in *Ctnnb1* were found in 14 out of 20 tumor samples (70%) showing polyclonality in this model.

### Blocker probes maximize Tm of the wild-type and mutant allele

**Figure 5:**
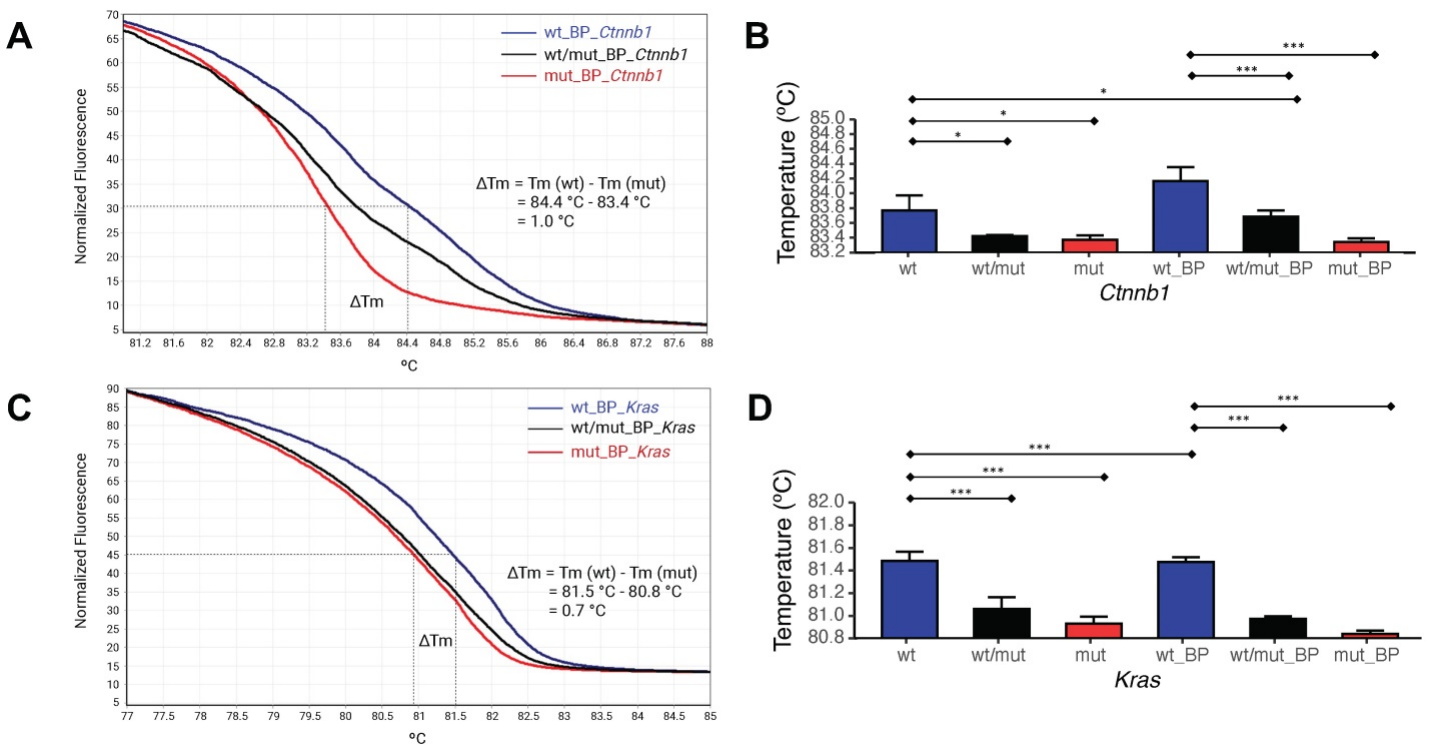
** Determination of the T_c_ for E-ice-COLD-PCR. (A)** HRMA for *Ctnnb1* using the wild type allele, mutant allele, and an equimolar mixture of both. **(B)** Statistical analysis of HRMA for *Ctnnb1*. **(C)** HRMA for *Kras* using the wild type allele, mutant allele, and an equimolar mixture of both. **(D)** Statistical analysis of HRMA for *Kras*. The experiment was carried out with three repetitions. The bars are presented as the mean ± SD analyzed by a one-way ANOVA followed by Tukey's multiple compare son test. *p < 0.05 and ***p < 0.001.

To enhance selective denaturalization and enrichment of mutant alleles by E-ice-COLD-PCR, the T_c_ of each of the BPs was determined by HRMA. T_c_ was defined as 84.4°C for the *Ctnnb1* wild-type allele BP and 83.4°C for the mutant allele BP, with Tm of 1.0°C between the two alleles (Figure 5A,B). The T_c_ for the wild-type allele BP of *Kras*, was in 81.5°C and for mutant allele BP was 80.8°C, with a Tm of 0.7°C (Figure 5C,D). Thus, the T_c_ for mutant enrichment of *Ctnnb1* and *Kras* was set at 83.4°C and 80.8°C, respectively.

### E-ice-COLD-PCR showed variable levels of enrichment in tumor samples and cfDNA

**Figure 6:**
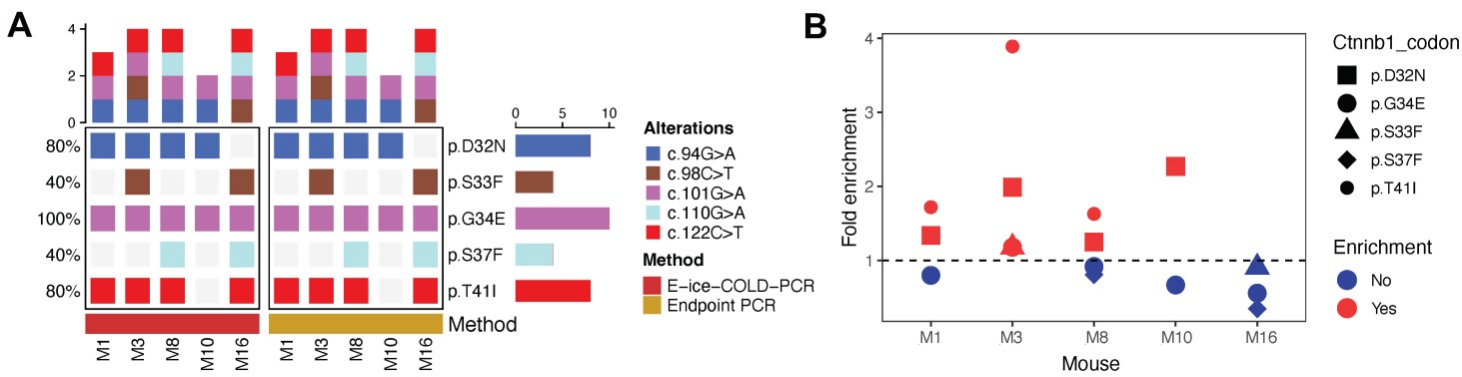
** Analysis of *Ctnnb1* mutations and fold enrichment in 5 tumor samples with E-ice-COLD-PCR and endpoint PCR. (A)** Mutations detected in *Ctnnb1*. Alterations are shown at the protein and cDNA level. **(B)** Fold enrichment in different codons of *Ctnnb1*. Fold enrichment = allelic fraction E-ice-COLD-PCR / allelic fraction endpoint PCR.

Prior to cfDNA analysis, we evaluated the sensitivity of E-ice-COLD-PCR and endpoint PCR followed by next generation sequencing (NGS) to detect mutations in *Ctnnb1* and *Kras* in 5 tumor samples (mice: M1, M3 M8, M10 and M16) previously analyzed with Sanger sequencing. We performed deep sequencing with a mean depth of 31,310X (±8,924) to characterize the *Kras* and *Ctnnb1* hotspot alleles in the tumor samples (Figure S3). We detected the same mutations in *Ctnnb1* with E-ice-COLD-PCR and endpoint PCR (Table S3, S5; Figure S4); *Kras* mutations were not detected by either approach (Figure 6A). We found different levels of enrichment with the two PCR techniques, mutations in *Ctnnb1* at codons 32 and 41 were preferentially enriched compared to codons 33, 34, and 37 (Figure 6B and Table S3). In contrast to Sanger Sequencing, endpoint PCR with NGS detected 5 additional mutations in 3 mice: M1 (codon 34), M3 (codon 33 and 34), and M16 (codon 33 and 37) (Figure 4 and 6A).

**Figure 7:**
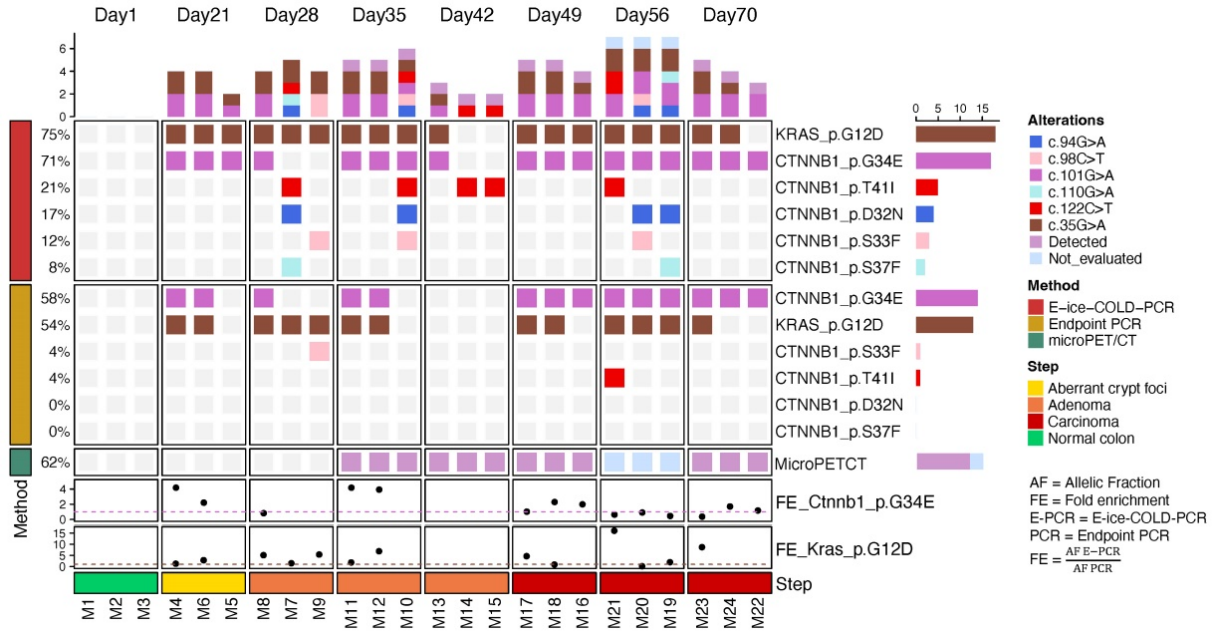
** Analysis of *Ctnnb1* and *Kras* mutations in cfDNA during development of CRC.** The oncoprint shows mutations detected in *Ctnnb1* and *Kras* with E-ice-COLD-PCR and endpoint PCR, tumor detection with microPET/CT, and fold enrichment of *Ctnnb1* at codon 32 and *Kras* at codon 12.

To detect mutations in cfDNA of the AOM/DSS group, we used the same strategies as for the tumor samples. Sequencing depth of cfDNA was 159,509X (±43,030) (Figure S3). E-ice-COLD-PCR detected 49 mutations in both genes, whereas endpoint PCR detected 29 mutations along the progression of the carcinogenic development (Figure 7; Tables S4, S6). Mutations in *Ctnnb1* were detected at codons 32, 33, 34, 37, and 41 with E-ice-COLD-PCR; endpoint PCR detected mutations at codons 33, 34, and 41. Both PCR techniques detected mutations in *Kras* at codon 12. E-ice-COLD-PCR revealed mutations in *Ctnnb1* and *Kras* in all the cfDNA samples analyzed (3/3), and endpoint PCR in 2 samples (2/3) at day 22, which corresponds to the formation of ACF, small precursor lesions for the development of CRC, which corresponds to an early detection. We identified different levels of enrichment, for instance *Ctnnb1* at codon 34 was preferentially enriched 2 to 4 times in the ACF, but not in advanced stage (Figure 7 and Table S4). *Kras,* on the other hand*,* was enriched in almost all stages of CRC. It was not possible to compare the levels of enrichment of *Ctnnb1* and *Kras* in some mice because they were detected only with E-ice-COLD-PCR. In addition, we identified polyclonality in *Ctnnb1* with E-ice-COLD-PCR in mouse M7 (day 28), mouse M10 (day 35); mice M19, M20, and M21 (day 56); and with endpoint PCR in mouse M21 (day 56).

## Discussion

Cancer is one of the leading causes of death worldwide. In 2020, WHO reported 935,173 deaths from CRC making it the second leading cause of cancer death in the world 1. Due to its asymptomatic course, late detection is a major factor contributing to CRC mortality. For decades, patient management with CRC has been based on colonoscopy, histopathological and molecular analysis of tumor tissues, computing tomography, magnetic resonance, and the evaluation of carcinoembryonic or carbohydrate antigens in serum. Despite their critical importance, these strategies still do not yet fully satisfy clinical needs due to their lack of sensitivity and/or specificity to detect early colorectal tumors 2-4. In contrast, the use of liquid biopsies such as cfDNA has gained interest due to its feasibility to detect tumor-specific mutations. However, there are some limitations to the use of cfDNA for early detection of CRC: i) due to the absence of samples in early stages of CRC development, several reports have used liquid biopsies for CRC management as a marker of tumor postoperative recurrence 5-7 and to assess the tumor burden in metastatic patients 8,9; ii) the tumor allelic fraction of cfDNA in early stages is low and it requires specialized molecular approaches such as BEAMing, digital PCR, and TAM-Seq 19,20. To address this problem, the purpose of this work was to detect somatic mutations associated with the early stage of CRC development using cfDNA in an *in vivo* model of induced carcinogenesis and by the implementation of a new molecular approach to identify tumor mutations with low allelic fractions.

**Figure 8:**
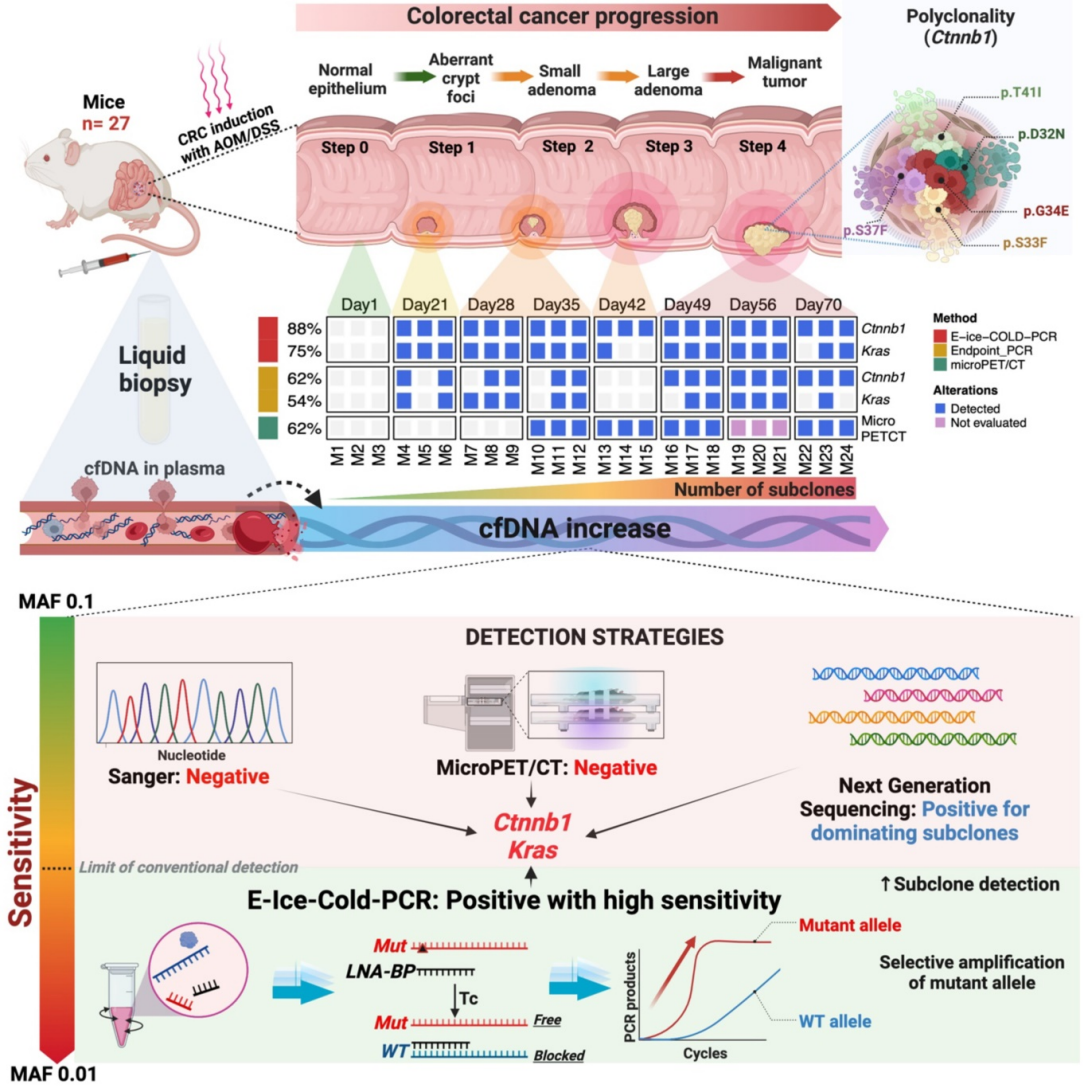
** Summary of key points for early detection of CRC using cfDNA.** At the top, the development of colon cancer and the use of liquid biopsy for tumor detection in the AOM/DSS model. At the bottom, the molecular and imaging techniques, as well as their sensitivity to detect CRC.

A well-established model of chemically induced carcinogenesis in mice was used to recreate the entire pathogenic process of CRC development 21,22, from its beginning to the locally advanced stage, as observed in humans. With this approach, it was possible to evaluate the molecular alterations in the early stage of CRC development since well-known hotspot mutations are generated in *Ctnnb1* and *Kras* (Figure 8). Before evaluating the genetic alterations, physiological changes were analyzed. The AOM/DSS group showed piloerection, diarrhea, bleeding, and anal protrusion, as well as loss of body weight after each cycle of DSS compared to the control group. These events are due to colonic epithelial cell damage and inflammation caused by DSS 23-25. On the other hand, the AOM/DSS group developed colonic tumors as previously described 10,22. Most of the tumors were located in the middle and distal colon, as is commonly observed in human CRC 26. Several studies have shown a correlation between cfDNA levels and tumor burden 27,28 and chronic inflammation 29,30. We observed an increase in the plasma concentration of cfDNA throughout tumor development. Thus, as reported in humans, tumor size and chronic inflammation induced in this CRC model influenced plasma cfDNA levels.

Radiology images are used in the clinic for accurate diagnosis and staging of CRC to make optimal therapeutic decisions for patients. Here, we used microPET/CT imaging to track the progression of mouse tumor growth in a clinically relevant setting. This strategy detected the tumor on day 35 in the AOM/DSS group. This model's time-point corresponds to the transition from microadenomas to adenomas, which are early events in the CRC development 21,22. The spatial resolution of microPET/CT is 1.5 mm; hence, smaller lesions are not detectable with this technique. The initial histopathological change of CRC is ACF, which is less than 1 mm in size; consequently, microPET/CT could not detect these lesions before day 35. In addition, on days 1, 35, and 42, 18FDG uptake was observed in brown fat because microPET/CT was taken in a climate-controlled room. Under low-temperature conditions, brown fat is activated for thermogenesis and does not represent a carcinogenic process as observed in this study. On days 49 and 70 of microPET/CT images, no 18FDG uptake was observed in brown fat because it was concentrated in the already established tumor mass.

Prior to cfDNA analysis, we evaluated the mutations with endpoint PCR and Sanger sequencing in the tumor samples of the AOM/DSS group. Mutations in *Ctnnb1* were found in all mice as previously reported 31,32. More than two different mutations in *Ctnnb1* were found in 14 out of 20 tumor samples, strongly suggesting polyclonality in this model, in which multiple subclones containing different mutant alleles coexist in the tumor. This phenomenon has been detected in human CRC, especially in familial adenomatous polyposis 33,34. This is the first time that this phenomenon is reported in the AOM/DSS model.

To detect somatic mutations associated to CRC development, we implemented a molecular approach to enrich and detect mutant alleles, designated E-ice-COLD-PCR, in the early stage of CRC development with cfDNA. E-ice-COLD-PCR has been used to detect somatic mutations in *BRAF* in fresh frozen tissue, FFPE, and plasma samples of melanoma patients 15; mutations in *KRAS* in fresh frozen tissues of CRC patients 14; and mutations in *KRAS* in cfDNA of metastatic CRC patients 35, but it has not been evaluated in the early stage of CCR development. To establish our early detection methodology, we first evaluated the mutations in tumor samples using E-ice-COLD-PCR and endpoint PCR, together with massively parallel sequencing. We detected the same mutations in both PCR techniques and the levels of enrichment were different in *Ctnnb1*. Mutations in *Ctnnb1* at codons 32 and 41 had a higher allelic fraction and enrichment with E-ice-COLD-PCR than with endpoint PCR (Figure 6; Table S3 and S5). We did not detect enrichment in* Ctnnb1* at codons 33, 34, and 37.

To detect *Ctnnb1* and *Kras* mutations in the cfDNA, we employed the same experimental strategy in the plasma sample. One of the major challenges in utilizing cfDNA is its low mutant allelic fraction, which is less than 0.1% in the early cancer stages. Therefore, we used the technique E-ice-COLD-PCR to enrich mutant alleles with a frequency of up to 0.01% 14,15. In this sense, the use of E-ice-COLD-PCR was not challenging in the tumor samples but was key in cfDNA, since it detected 49 mutations compared to endpoint PCR, which detected only 29 mutations in the cfDNA of the AOM/DSS group (Figure 7; Table S4 and S6). At the earliest step of CRC development, the formation of ACF (day 22), which is only identifiable at the histological level, we detected mutations in *Ctnnb1* and *Kras* in all the mice analyzed (3/3) with E-ice-COLD-PCR, and in two mice (2/3) with endpoint PCR. In comparison to microPET/CT, which revealed the establishment of microadenoma until day 35, the mutational analysis of cfDNA allowed earlier detection, up to 10 days sooner. This period corresponds to about 5-10 years in human patients, which represents a real advantage in the detection of this disease 36. The discovery of more mutations at the ACF formation stage with the use of E-ice-COLD-PCR compared to endpoint PCR, was an important result. This could favor the use of E-ice-COLD-PCR for the early detection of CRC. On the other hand, the analysis of cfDNA was able to capture polyclonality at different steps of CRC formation, which could enable the potential use of liquid biopsy to capture the intratumoral heterogeneity in CRC.

In this study, two blocker probes with LNA were designed for E-ice-COLD-PCR: one to enrich mutation in *Ctnnb1* at codons 32, 33, 34, 37 and 41, and one for *Kras* at codon 12. We reasoned that this strategy would enhance the enrichment of all five hotspot mutations in *Ctnnb1* with a single probe. However, the addition of LNAs decreased the enrichment of some mutations regardless of the tumor development stage. This drawback could be resolved by designing specific probes with LNAs for each specific mutation. In contrast, the use of one BP to detect mutations in *Kras* using cfDNA was favorable since it detected 5 more mutations than endpoint PCR. Enrichment in *Kras* was observed at different steps of tumor development.

In clinical practice, the use of tumor DNA obtained from tissue biopsies is considered the gold standard for genotyping and directed diagnosis. Nevertheless, these practices are always associated with complications due to surgical procedures and in some patients the tumor sample is inaccessible. Another problem with the use of biopsies is the limited size of the tissues, which does not capture the full clonal heterogeneity present in the tumor, resulting in false negative results, incomplete genotyping and ultimately the selection of ineffective therapies. On the other hand, the analysis of cfDNA in the form of a non-invasive liquid biopsy, better captures the subclonal heterogeneity and provides the same molecular information present in the tumor sample, and it is a sample easy to obtain in medical consultations.

As a proof of principle, this work demonstrates that the selective enrichment strategy of mutant alleles by E-ice-COLD-PCR coupled to parallel massive sequencing can be used to improve the detection of CRC using liquid biopsies, even in the earliest stages of neoplasia, while conventional techniques such as advanced imaging does not allow the detection of the disease. However, we acknowledge that our study is limited by: i) the small sample number of mice evaluated at each stage of the disease progression, which prevented a more detailed analysis, as well as the absence of a rigorous evaluation of the sensitivity and specificity of the method; ii) we could not validate our findings with other external molecular methods such as digital PCR or BEAMing; and iii) our inability to validate of our molecular design in an independent cohort with mice or humans. Nevertheless, in the absence of other molecular methods, we used microPET/CT to validate our molecular findings and have a biological context analogous to a clinical scenario for humans. The primers and BPs were designed specifically for the mouse alleles. However, the robustness of this method could be transferred to the clinic with a design directed to human alleles associated with CRC.

## Conclusion

In this work, we demonstrate the feasibility of using cfDNA as a liquid biopsy for mutation detection in cfDNA as a non-invasive technique in the earliest stages of CRC in a murine model by allele-specific PCR. Allele-specific PCR coupled with massive sequencing allowed earlier detection of CRC compared to PET/CT imaging tests, which demonstrated the proof of principle. The results of this work provide scientific evidence for establishing methodological bases that allow the early detection of CRC in humans by liquid biopsy molecular analysis.

## Supplementary Material

Supplementary figures and tables.

## Abbreviations

CRC: Colorectal cancer
cfDNA: circulating free DNA
E-ice-COLD-PCR: Enhanced-improved and Complete Enrichment CO-amplification at Lower Denaturation temperature Polymerase Chain Reaction
microPET/CT: micro positron emission tomography/computed tomography
ACF: aberrant crypt foci
AOM: azoxymethane
DSS: dextran sulfate sodium
18FDG: 2-[18F]-fluoro-2-deoxy-D-glucosa
TLG: total lesion glycolysis
BP: Blocker probe
LNA: locked nucleic acid
Tm: melting temperature
Tc: critical temperature
NGS: next generation sequencing
HRMA: high resolution melting analysis
