# Supplementary material for: Early detection of colorectal cancer somatic mutations using cfDNA liquid biopsies in a murine carcinogenesis model
Source: J Cancer. 2022 Sep 25;13(13):3404–14. doi: 10.7150/jca.76516 (PMC9608204; doi:10.7150/jca.76516)
Supplement: Supplementary file 1 — Supplementary figures and tables. [file jcav13p3404s1.pdf]

# Supplementary materials

**Supp. Table 1 (S1)**
Primers used to amplify *Ctnnb1* and *Kras* for Endpoint PCR and E-ice-COLD-PCR

| Primer | Name | Length | Tm | Sequence | Product |
|---|---|---|---|---|---|
| Forward | Ctnnb1 | 20 | 60,37 | CACTGGCAGCAGCAGTCTTA | 111 |
| Reverse | | 22 | 59,98 | GGAGGTGTCAACATCTTCTTCC | |
| Forward | Kras | 20 | 58,46 | AGGCCTGCTGAAAATGACTG | 135 |
| Reverse | | 23 | 61,61 | AGCAGCGTTACCTCTATCGTAGG | |

**Supp. Table 2 (S2)**
Blocker probes used to *Ctnnb1* and *Kras* in E-ice-COLD-PCR

| Probe | Sequence |
|---|---|
| **Ctnnb1_BP_41** | TCTTACTTG+G+AT+T+C+TG+G+AATCCAT+T+C+TGGTGCCACCA+C+C+AC/3Phos |
| **Kras_BP_42** | GACTGAGTATAAACTTGTGGTGGTTGGAGCTG+G+TGGCGTAGG/3Phos |
| **Locked nucleic acid (LNA): +A, +G, +C , and +T** | |

**Supp. Table 3 (S3)**
Mutations detected in the tumor samples with E-ice-COLD-PCR and endpoint PCR together with massive parallel sequencing

| ID | Method | Gene | Type of change | Transcript | Exon | cfDNA change | Protein change | Allelic fraction |
|---|---|---|---|---|---|---|---|---|
| **EPCR1** | E-PCR | Ctnnb1 | nonsynonymous SNV | NM_007614.3 | 3 | c.94G>A | p.D32N | 18.44 |
| | | | | | | c.101G>A | p.G34E | 2.04 |
| | | | | | | c.122C>T | p.T41I | 19.92 |
| **EPCR3** | E-PCR | *Ctnnb1* | nonsynonymous SNV | NM_007614.3 | 3 | c.94G>A | p.D32N | 22.69 |
| | | | | | | c.98C>T | p.S33F | 2.85 |
| | | | | | | c.101G>A | p.G34E | 3.14 |
| | | | | | | c.122C>T | p.T41I | 13.4 |
| **EPCR8** | E-PCR | *Ctnnb1* | nonsynonymous SNV | NM_007614.3 | 3 | c.94G>A | p.D32N | 5.84 |
| | | | | | | c.101G>A | p.G34E | 3.22 |
| | | | | | | c.110C>T | p.S37F | 9.05 |
| | | | | | | c.122C>T | p.T41I | 11.79 |
| **EPCR10** | E-PCR | *Ctnnb1* | nonsynonymous SNV | NM_007614.3 | 3 | c.94G>A | p.D32N | 22.43 |
| | | | | | | c.101G>A | p.G34E | 9.93 |
| **EPCR16** | E-PCR | *Ctnnb1* | nonsynonymous SNV | NM_007614.3 | 3 | c.98C>T | p.S33F | 2.33 |
| | | | | | | c.101G>A | p.G34E | 5.39 |
| | | | | | | c.110C>T | p.S37F | 1.0 |
| | | | | | | c.122C>T | p.T41I | 5.57 |
| **PCR1** | PCR | *Ctnnb1* | nonsynonymous SNV | NM_007614.3 | 3 | c.94G>A | p.D32N | 13.71 |
| | | | | | | c.101G>A | p.G34E | 2.54 |
| | | | | | | c.122C>T | p.T41I | 11.53 |
| **PCR3** | PCR | *Ctnnb1* | nonsynonymous SNV | NM_007614.3 | 3 | c.94G>A | p.D32N | 11.37 |
| | | | | | | c.98C>T | p.S33F | 2.4 |
| | | | | | | c.101G>A | p.G34E | 2.65 |
| | | | | | | c.122C>T | p.T41I | 3.44 |
| **PCR8** | PCR | *Ctnnb1* | nonsynonymous SNV | NM_007614.3 | 3 | c.94G>A | p.D32N | 4.38 |
| | | | | | | c.101G>A | p.G34E | 3.47 |
| | | | | | | c.110C>T | p.S37F | 11.16 |
| | | | | | | c.122C>T | p.T41I | 7.21 |
| **PCR10** | PCR | *Ctnnb1* | nonsynonymous SNV | NM_007614.3 | 3 | c.94G>A | p.D32N | 9.85 |
| | | | | | | c.101G>A | p.G34E | 14.67 |
| **PCR16** | PCR | *Ctnnb1* | nonsynonymous SNV | NM_007614.3 | 3 | c.98C>T | p.S33F | 2.56 |
| | | | | | | c.101G>A | p.G34E | 9.58 |
| | | | | | | c.110C>T | p.S37F | 2.84 |
| | | | | | | c.122C>T | p.T41I | 5.95 |

PCR: Endpoint PCR, E-PCR: E-ice-COLD-PCR

**Supp. Table 4 (S4)**

Mutations detected in the cfDNA samples with E-ice-COLD-PCR and endpoint PCR together with massive parallel sequencing

| Day | Step | ID | Method | Gene | Type of change | Transcript | Exon | cfDNA change | Protein change | Allelic fraction |
|---|---|---|---|---|---|---|---|---|---|---|
| **01** | Normal | M1<br>M2<br>M3 | E-PCR<br>E-PCR<br>E-PCR | ND | ND | ND | ND | ND | ND | ND |
| | | M1<br>M2<br>M3 | PCR<br>PCR<br>PCR | ND | ND | ND | ND | ND | ND | ND |
| **21** | ACF | M4 | E-PCR | *Ctnnb1* | nonsynonymous SNV | NM_007614.3 | 3 | c.101G>A | p.G34E | 9.66 |
| | | | | *Kras* | nonsynonymous SNV | NM_021284.6 | 2 | c.35G>A | p.G12D | 12.32 |
| | | M5 | E-PCR | *Ctnnb1* | nonsynonymous SNV | NM_007614.3 | 3 | c.101G>A | p.G34E | 3.67 |
| | | | | *Kras* | nonsynonymous SNV | NM_021284.6 | 2 | c.35G>A | p.G12D | 12.05 |
| | | M6 | E-PCR | *Ctnnb1* | nonsynonymous SNV | NM_007614.3 | 3 | c.101G>A | p.G34E | 7.55 |
| | | | | *Kras* | nonsynonymous SNV | NM_021284.6 | 2 | c.35G>A | p.G12D | 34.75 |
| | | M4 | PCR | *Ctnnb1* | nonsynonymous SNV | NM_007614.3 | 3 | c.101G>A | p.G34E | 2.31 |
| | | | | *Kras* | nonsynonymous SNV | NM_021284.6 | 2 | c.35G>A | p.G12D | 9.65 |
| | | M5 | PCR | ND | ND | ND | ND | ND | ND | ND |
| | | M6 | PCR | *Ctnnb1* | nonsynonymous SNV | NM_021284.6 | 3 | c.101G>A | p.G34E | 3.42 |
| | | | | *Kras* | nonsynonymous SNV | NM_007614.3 | 2 | c.35G>A | p.G12D | 12.23 |
| **28** | Adenoma | M7 | E-PCR | *Ctnnb1* | nonsynonymous SNV | NM_021284.6 | 3 | c.94G>A | p.D32N | 8.9 |
| | | | | *Ctnnb1* | nonsynonymous SNV | NM_021284.6 | 3 | c.98C>T | p.S33F | 8.6 |
| | | | | *Ctnnb1* | nonsynonymous SNV | NM_021284.6 | 3 | c.110C>T | p.S37F | 2 |
| | | | | *Ctnnb1* | nonsynonymous SNV | NM_021284.6 | 3 | c.122C>T | p.T41I | 1.99 |
| | | | | *Kras* | nonsynonymous SNV | NM_021284.6 | 2 | c.35G>A | p.G12D | 19.56 |
| | | M8 | E-PCR | *Ctnnb1* | nonsynonymous SNV | NM_021284.6 | 3 | c.101G>A | p.G34E | 2.48 |
| | | | | *Kras* | nonsynonymous SNV | NM_021284.6 | 2 | c.35G>A | p.G12D | 28.4 |
| | | M9 | E-PCR | *Ctnnb1* | nonsynonymous SNV | NM_021284.6 | 3 | c.98C>T | p.S33F | 2.39 |
| | | | | *Kras* | nonsynonymous SNV | NM_021284.6 | 2 | c.35G>A | p.G12D | 29.08 |
| | | M7 | PCR | *Kras* | nonsynonymous SNV | NM_021284.6 | 2 | c.35G>A | p.G12D | 13.14 |
| | | M8 | PCR | *Ctnnb1* | nonsynonymous SNV | NM_021284.6 | 3 | c.101G>A | p.G34E | 3.05 |
| | | | | *Kras* | nonsynonymous SNV | NM_021284.6 | 2 | c.35G>A | p.G12D | 5.56 |
| | | M9 | PCR | *Ctnnb1* | nonsynonymous SNV | NM_021284.6 | 3 | c.98C>T | p.S33F | 4.14 |
| | | | | *Kras* | nonsynonymous SNV | NM_021284.6 | 2 | c.35G>A | p.G12D | 5.39 |
| **35** | Adenoma | M10 | E-PCR | *Ctnnb1* | nonsynonymous SNV | NM_021284.6 | 3 | c.94G>A | p.D32N | 7.68 |
| | | | | *Ctnnb1* | nonsynonymous SNV | NM_021284.6 | 3 | c.98C>T | p.S33F | 1.6 |
| | | | | *Ctnnb1* | nonsynonymous SNV | NM_021284.6 | 3 | c.101G>A | p.G34E | 1.05 |
| | | | | *Ctnnb1* | nonsynonymous SNV | NM_021284.6 | 3 | c.122C>T | p.T41I | 6.59 |
| | | | | *Kras* | nonsynonymous SNV | NM_021284.6 | 2 | c.35G>A | p.G12D | 36.36 |

| | | | | | | | | | | |
|---|---|---|---|---|---|---|---|---|---|---|
| | | M11 | E-PCR | *Ctnnb1* | nonsynonymous SNV | NM_021284.6 | 3 | c.101G>A | p.G34E | 11.7 |
| | | | | *Kras* | nonsynonymous SNV | NM_021284.6 | 2 | c.35G>A | p.G12D | 50.4 |
| | | M12 | E-PCR | *Ctnnb1* | nonsynonymous SNV | NM_021284.6 | 3 | c.101G>A | p.G34E | 27.37 |
| | | | | *Kras* | nonsynonymous SNV | NM_021284.6 | 2 | c.35G>A | p.G12D | 54.54 |
| | | M10 | PCR | *ND* | ND | ND | ND | ND | ND | ND |
| | | M11 | PCR | *Ctnnb1* | nonsynonymous SNV | NM_021284.6 | 3 | c.101G>A | p.G34E | 2.8 |
| | | | | *Kras* | nonsynonymous SNV | NM_021284.6 | 2 | c.35G>A | p.G12D | 27.47 |
| | | M12 | PCR | *Ctnnb1* | nonsynonymous SNV | NM_021284.6 | 3 | c.101G>A | p.G34E | 6.97 |
| | | | | *Kras* | nonsynonymous SNV | NM_021284.6 | 2 | c.35G>A | p.G12D | 7.92 |
| **42** | Adenoma | M13 | E-PCR | *Ctnnb1* | nonsynonymous SNV | NM_021284.6 | 3 | c.101G>A | p.G34E | 25.18 |
| | | | | *Kras* | nonsynonymous SNV | NM_021284.6 | 2 | c.35G>A | p.G12D | 43.4 |
| | | M14 | E-PCR | *Ctnnb1* | nonsynonymous SNV | NM_021284.6 | 3 | c.122C>T | p.T41I | 20.71 |
| | | M15 | E-PCR | *Ctnnb1* | nonsynonymous SNV | NM_021284.6 | 3 | c.122C>T | p.T41I | 20.81 |
| | | M13 | PCR | ND | ND | ND | ND | ND | ND | ND |
| | | M14 | PCR | ND | ND | ND | ND | ND | ND | ND |
| | | M15 | PCR | ND | ND | ND | ND | ND | ND | ND |
| **49** | Carcinoma | M16 | E-PCR | *Ctnnb1* | nonsynonymous SNV | NM_021284.6 | 3 | c.101G>A | p.G34E | 8.7 |
| | | | | *Kras* | nonsynonymous SNV | NM_021284.6 | 2 | c.35G>A | p.G12D | 59.72 |
| | | M17 | E-PCR | *Ctnnb1* | nonsynonymous SNV | NM_021284.6 | 3 | c.101G>A | p.G34E | 6.59 |
| | | | | *Kras* | nonsynonymous SNV | NM_021284.6 | 2 | c.35G>A | p.G12D | 60.79 |
| | | M18 | E-PCR | *Ctnnb1* | nonsynonymous SNV | NM_021284.6 | 3 | c.101G>A | p.G34E | 15.37 |
| | | | | *Kras* | nonsynonymous SNV | NM_021284.6 | 2 | c.35G>A | p.G12D | 20.65 |
| | | M16 | PCR | *Ctnnb1* | nonsynonymous SNV | NM_021284.6 | 3 | c.101G>A | p.G34E | 4.38 |
| | | M17 | PCR | *Ctnnb1* | nonsynonymous SNV | NM_021284.6 | 3 | c.101G>A | p.G34E | 6.489 |
| | | | | *Kras* | nonsynonymous SNV | NM_021284.6 | 2 | c.35G>A | p.G12D | 13.07 |
| | | M18 | PCR | *Ctnnb1* | nonsynonymous SNV | NM_021284.6 | 3 | c.101G>A | p.G34E | 6.7 |
| | | | | *Kras* | nonsynonymous SNV | NM_021284.6 | 2 | c.35G>A | p.G12D | 23.34 |
| **56** | Carcinoma | M19 | E-PCR | *Ctnnb1* | nonsynonymous SNV | NM_021284.6 | 3 | c.94G>A | p.D32N | 1.43 |
| | | | | *Ctnnb1* | nonsynonymous SNV | NM_021284.6 | 3 | c.101G>A | p.G34E | 1.66 |
| | | | | *Ctnnb1* | nonsynonymous SNV | NM_021284.6 | 3 | c.110C>T | p.S37F | 1.37 |
| | | | | *Kras* | nonsynonymous SNV | NM_021284.6 | 2 | c.35G>A | p.G12D | 10.12 |
| | | M20 | E-PCR | *Ctnnb1* | nonsynonymous SNV | NM_021284.6 | 3 | c.94G>A | p.D32N | 6.8 |
| | | | | *Ctnnb1* | nonsynonymous SNV | NM_021284.6 | 3 | c.98C>T | p.S33F | 2.11 |
| | | | | *Ctnnb1* | nonsynonymous SNV | NM_021284.6 | 3 | c.101G>A | p.G34E | 3.18 |
| | | | | *Kras* | nonsynonymous SNV | NM_021284.6 | 2 | c.35G>A | p.G12D | 1.23 |
| | | M21 | E-PCR | *Ctnnb1* | nonsynonymous SNV | NM_021284.6 | 3 | c.101G>A | p.G34E | 2.23 |
| | | | | *Ctnnb1* | nonsynonymous SNV | NM_021284.6 | 3 | c.110C>T | p.S37F | 1.4 |
| | | | | *Kras* | nonsynonymous SNV | NM_021284.6 | 2 | c.35G>A | p.G12D | 35.49 |
| | | M19 | PCR | *Ctnnb1* | nonsynonymous SNV | NM_021284.6 | 3 | c.101G>A | p.G34E | 3.68 |

| | | | | | | | | | | |
|---|---|---|---|---|---|---|---|---|---|---|
| | | | | *Kras* | nonsynonymous SNV | NM_021284.6 | 2 | c.35G>A | p.G12D | 5.19 |
| | | M20 | PCR | *Ctnnb1* | nonsynonymous SNV | NM_021284.6 | 3 | c.101G>A | p.G34E | 3.51 |
| | | | | *Kras* | nonsynonymous SNV | NM_021284.6 | 2 | c.35G>A | p.G12D | 7.68 |
| | | M21 | PCR | *Ctnnb1* | nonsynonymous SNV | NM_021284.6 | 3 | c.101G>A | p.G34E | 3.55 |
| | | | | *Kras* | nonsynonymous SNV | NM_021284.6 | 2 | c.35G>A | p.G12D | 2.21 |
| **70** | Carcinoma | M22 | E-PCR | *Ctnnb1* | nonsynonymous SNV | NM_021284.6 | 3 | c.101G>A | p.G34E | 3.4 |
| | | M23 | E-PCR | *Ctnnb1* | nonsynonymous SNV | NM_021284.6 | 3 | c.101G>A | p.G34E | 1.0 |
| | | | | *Kras* | nonsynonymous SNV | NM_021284.6 | 2 | c.35G>A | p.G12D | 18.91 |
| | | M24 | E-PCR | *Ctnnb1* | nonsynonymous SNV | NM_021284.6 | 3 | c.101G>A | p.G34E | 11.04 |
| | | | | *Kras* | nonsynonymous SNV | NM_021284.6 | 2 | c.35G>A | p.G12D | 3 |
| | | M22 | PCR | *Ctnnb1* | nonsynonymous SNV | NM_021284.6 | 3 | c.101G>A | p.G34E | 2.9 |
| | | M23 | PCR | *Ctnnb1* | nonsynonymous SNV | NM_021284.6 | 3 | c.101G>A | p.G34E | 2.8 |
| | | | | *Kras* | nonsynonymous SNV | NM_021284.6 | 2 | c.35G>A | p.G12D | 2.17 |
| | | M24 | PCR | *Ctnnb1* | nonsynonymous SNV | NM_021284.6 | 3 | c.101G>A | p.G34E | 6.48 |

PCR: Endpoint PCR, E-PCR: E-ice-COLD-PCR

**Supp. Table 5 (S5)**

Mutations detected in the tumor samples with endpoint PCR together with massive parallel sequencing

| Sample | ID | Chr | Star | REF | ALT | Func.refGene | Gene.refGene | ExonicFunc.refGene | AAChange.refGene | GT | AF | DP |
|---|---|---|---|---|---|---|---|---|---|---|---|---|
| Tumor | EPCR1 | chr9 | 120950602 | G | A | exonic | CTNNB1 | nonsynonymous SNV | CTNNB1:NM_007614:exon3:c.G94A:p.D32N | 0/1 | 0.1844 | 491 |
| Tumor | EPCR1 | chr9 | 120950609 | G | A | exonic | CTNNB1 | nonsynonymous SNV | CTNNB1:NM_007614:exon3:c.G101A:p.G34E | 0/1 | 0.0204 | 499 |
| Tumor | EPCR1 | chr9 | 120950630 | C | T | exonic | CTNNB1 | nonsynonymous SNV | CTNNB1:NM_007614:exon3:c.C122T:p.T41I | 0/1 | 0.1992 | 505 |
| Tumor | EPCR3 | chr9 | 120950602 | G | A | exonic | CTNNB1 | nonsynonymous SNV | CTNNB1:NM_007614:exon3:c.G94A:p.D32N | 0/1 | 0.2269 | 411 |
| Tumor | EPCR3 | chr9 | 120950606 | C | T | exonic | CTNNB1 | nonsynonymous SNV | CTNNB1:NM_007614:exon3:c.C98T:p.S33F | 0/1 | 0.0285 | 420 |
| Tumor | EPCR3 | chr9 | 120950609 | G | A | exonic | CTNNB1 | nonsynonymous SNV | CTNNB1:NM_007614:exon3:c.G101A:p.G34E | 0/1 | 0.0314 | 419 |
| Tumor | EPCR3 | chr9 | 120950630 | C | T | exonic | CTNNB1 | nonsynonymous SNV | CTNNB1:NM_007614:exon3:c.C122T:p.T41I | 0/1 | 0.134 | 430 |
| Tumor | EPCR8 | chr9 | 120950602 | G | A | exonic | CTNNB1 | nonsynonymous SNV | CTNNB1:NM_007614:exon3:c.G94A:p.D32N | 0/1 | 0.0548 | 433 |
| Tumor | EPCR8 | chr9 | 120950609 | G | A | exonic | CTNNB1 | nonsynonymous SNV | CTNNB1:NM_007614:exon3:c.G101A:p.G34E | 0/1 | 0.0322 | 445 |
| Tumor | EPCR8 | chr9 | 120950618 | C | T | exonic | CTNNB1 | nonsynonymous SNV | CTNNB1:NM_007614:exon3:c.C110T:p.S37F | 0/1 | 0.0905 | 456 |
| Tumor | EPCR8 | chr9 | 120950630 | C | T | exonic | CTNNB1 | nonsynonymous SNV | CTNNB1:NM_007614:exon3:c.C122T:p.T41I | 0/1 | 0.1179 | 456 |
| Tumor | EPCR10 | chr9 | 120950602 | G | A | exonic | CTNNB1 | nonsynonymous SNV | CTNNB1:NM_007614:exon3:c.G94A:p.D32N | 0/1 | 0.2243 | 374 |
| Tumor | EPCR10 | chr9 | 120950609 | G | A | exonic | CTNNB1 | nonsynonymous SNV | CTNNB1:NM_007614:exon3:c.G101A:p.G34E | 0/1 | 0.0993 | 383 |
| Tumor | EPCR16 | chr9 | 120950606 | C | T | exonic | CTNNB1 | nonsynonymous SNV | CTNNB1:NM_007614:exon3:c.C98T:p.S33F | 0/1 | 0.0233 | 457 |
| Tumor | EPCR16 | chr9 | 120950609 | G | A | exonic | CTNNB1 | nonsynonymous SNV | CTNNB1:NM_007614:exon3:c.G101A:p.G34E | 0/1 | 0.0539 | 436 |
| Tumor | EPCR16 | chr9 | 120950618 | C | T | exonic | CTNNB1 | nonsynonymous SNV | CTNNB1:NM_007614:exon3:c.C110T:p.S37F | 0/1 | 0.01 | 446 |
| Tumor | EPCR16 | chr9 | 120950630 | C | T | exonic | CTNNB1 | nonsynonymous SNV | CTNNB1:NM_007614:exon3:c.C122T:p.T41I | 0/1 | 0.0557 | 441 |

| | | | | | | | | | | | | |
|---|---|---|---|---|---|---|---|---|---|---|---|---|
| Tumor | PCR1 | chr9 | 120950602 | G | A | exonic | CTNNB1 | nonsynonymous SNV | CTNNB1:NM_007614:exon3:c.G94A:p.D32N | 0/1 | 0.1371 | 467 |
| Tumor | PCR1 | chr9 | 120950609 | G | A | exonic | CTNNB1 | nonsynonymous SNV | CTNNB1:NM_007614:exon3:c.G101A:p.G34E | 0/1 | 0.0254 | 479 |
| Tumor | PCR1 | chr9 | 120950630 | C | T | exonic | CTNNB1 | nonsynonymous SNV | CTNNB1:NM_007614:exon3:c.C122T:p.T41I | 0/1 | 0.1153 | 483 |
| Tumor | PCR3 | chr9 | 120950602 | G | A | exonic | CTNNB1 | nonsynonymous SNV | CTNNB1:NM_007614:exon3:c.G94A:p.D32N | 0/1 | 0.1137 | 440 |
| Tumor | PCR3 | chr9 | 120950606 | C | T | exonic | CTNNB1 | nonsynonymous SNV | CTNNB1:NM_007614:exon3:c.C98T:p.S33F | 0/1 | 0.024 | 436 |
| Tumor | PCR3 | chr9 | 120950609 | G | A | exonic | CTNNB1 | nonsynonymous SNV | CTNNB1:NM_007614:exon3:c.G101A:p.G34E | 0/1 | 0.0265 | 459 |
| Tumor | PCR3 | chr9 | 120950630 | C | T | exonic | CTNNB1 | nonsynonymous SNV | CTNNB1:NM_007614:exon3:c.C122T:p.T41I | 0/1 | 0.0344 | 465 |
| Tumor | PCR8 | chr9 | 120950602 | G | A | exonic | CTNNB1 | nonsynonymous SNV | CTNNB1:NM_007614:exon3:c.G94A:p.D32N | 0/1 | 0.0438 | 501 |
| Tumor | PCR8 | chr9 | 120950609 | G | A | exonic | CTNNB1 | nonsynonymous SNV | CTNNB1:NM_007614:exon3:c.G101A:p.G34E | 0/1 | 0.0347 | 506 |
| Tumor | PCR8 | chr9 | 120950618 | C | T | exonic | CTNNB1 | nonsynonymous SNV | CTNNB1:NM_007614:exon3:c.C110T:p.S37F | 0/1 | 0.1116 | 513 |
| Tumor | PCR8 | chr9 | 120950630 | C | T | exonic | CTNNB1 | nonsynonymous SNV | CTNNB1:NM_007614:exon3:c.C122T:p.T41I | 0/1 | 0.0721 | 519 |
| Tumor | PCR10 | chr9 | 120950602 | G | A | exonic | CTNNB1 | nonsynonymous SNV | CTNNB1:NM_007614:exon3:c.G94A:p.D32N | 0/1 | 0.0985 | 507 |
| Tumor | PCR10 | chr9 | 120950609 | G | A | exonic | CTNNB1 | nonsynonymous SNV | CTNNB1:NM_007614:exon3:c.G101A:p.G34E | 0/1 | 0.1467 | 521 |
| Tumor | PCR16 | chr9 | 120950606 | C | T | exonic | CTNNB1 | nonsynonymous SNV | CTNNB1:NM_007614:exon3:c.C98T:p.S33F | 0/1 | 0.0256 | 543 |
| Tumor | PCR16 | chr9 | 120950609 | G | A | exonic | CTNNB1 | nonsynonymous SNV | CTNNB1:NM_007614:exon3:c.G101A:p.G34E | 0/1 | 0.0958 | 547 |
| Tumor | PCR16 | chr9 | 120950618 | C | T | exonic | CTNNB1 | nonsynonymous SNV | CTNNB1:NM_007614:exon3:c.C110T:p.S37F | 0/1 | 0.0284 | 565 |
| Tumor | PCR16 | chr9 | 120950630 | C | T | exonic | CTNNB1 | nonsynonymous SNV | CTNNB1:NM_007614:exon3:c.C122T:p.T41I | 0/1 | 0.0595 | 564 |

**Supp. Table 6 (S6)**

Mutations detected in the cfDNA samples with E-ice-COLD-PCR and endpoint PCR together with massive parallel sequencing

| Sample | ID | Chr | Star | REF | ALT | Func.refGene | Gene.refGene | ExonicFunc.refGene | AAChange.refGene | GT | AF | DP |
|---|---|---|---|---|---|---|---|---|---|---|---|---|
| cfDNA | EPCR4 | chr6 | 145246771 | C | T | exonic | KRAS | nonsynonymous SNV | KRAS:NM_021284:exon2:c.C35T:p.G12D | 0/1 | 0.1232 | 1437 |
| cfDNA | EPCR4 | chr9 | 120950609 | G | A | exonic | CTNNB1 | nonsynonymous SNV | CTNNB1:NM_007614:exon3:c.G101A:p.G34E | 0/1 | 0.0966 | 863 |
| cfDNA | EPCR5 | chr6 | 145246771 | C | T | exonic | KRAS | nonsynonymous SNV | KRAS:NM_021284:exon2:c.C35T:p.G12D | 0/1 | 0.1205 | 1780 |
| cfDNA | EPCR5 | chr9 | 120950609 | G | A | exonic | CTNNB1 | nonsynonymous SNV | CTNNB1:NM_007614:exon3:c.G101A:p.G34E | 0/1 | 0.0367 | 1063 |
| cfDNA | EPCR6 | chr6 | 145246771 | C | T | exonic | KRAS | nonsynonymous SNV | KRAS:NM_021284:exon2:c.C35T:p.G12D | 0/1 | 0.3475 | 1127 |
| cfDNA | EPCR6 | chr9 | 120950609 | G | A | exonic | CTNNB1 | nonsynonymous SNV | CTNNB1:NM_007614:exon3:c.G101A:p.G34E | 0/1 | 0.0755 | 1292 |
| cfDNA | EPCR7 | chr6 | 145246771 | C | T | exonic | KRAS | nonsynonymous SNV | KRAS:NM_021284:exon2:c.C35T:p.G12D | 0/1 | 0.1956 | 1806 |
| cfDNA | EPCR7 | chr9 | 120950602 | G | A | exonic | CTNNB1 | nonsynonymous SNV | CTNNB1:NM_007614:exon3:c.G94A:p.D32N | 0/1 | 0.089 | 808 |
| cfDNA | EPCR7 | chr9 | 120950606 | C | T | exonic | CTNNB1 | nonsynonymous SNV | CTNNB1:NM_007614:exon3:c.C98T:p.S33F | 0/1 | 0.086 | 925 |
| cfDNA | EPCR7 | chr9 | 120950618 | C | T | exonic | CTNNB1 | nonsynonymous SNV | CTNNB1:NM_007614:exon3:c.C110T:p.S37F | 0/1 | 0.02 | 987 |
| cfDNA | EPCR7 | chr9 | 120950630 | C | T | exonic | CTNNB1 | nonsynonymous SNV | CTNNB1:NM_007614:exon3:c.C122T:p.T41I | 0/1 | 0.019 | 803 |
| cfDNA | EPCR8 | chr6 | 145246771 | C | T | exonic | KRAS | nonsynonymous SNV | KRAS:NM_021284:exon2:c.C35T:p.G12D | 0/1 | 0.284 | 1072 |
| cfDNA | EPCR8 | chr9 | 120950609 | G | A | exonic | CTNNB1 | nonsynonymous SNV | CTNNB1:NM_007614:exon3:c.G101A:p.G34E | 0/1 | 0.0239 | 1042 |
| cfDNA | EPCR9 | chr6 | 145246771 | C | T | exonic | KRAS | nonsynonymous SNV | KRAS:NM_021284:exon2:c.C35T:p.G12D | 0/1 | 0.2908 | 1828 |
| cfDNA | EPCR9 | chr9 | 120950606 | C | T | exonic | CTNNB1 | nonsynonymous SNV | CTNNB1:NM_007614:exon3:c.C98T:p.S33F | 0/1 | 0.0239 | 758 |
| cfDNA | EPCR10 | chr6 | 145246771 | C | T | exonic | KRAS | nonsynonymous SNV | KRAS:NM_021284:exon2:c.C35T:p.G12D | 0/1 | 0.3636 | 1483 |
| cfDNA | EPCR10 | chr9 | 120950602 | G | A | exonic | CTNNB1 | nonsynonymous SNV | CTNNB1:NM_007614:exon3:c.G94A:p.D32N | 0/1 | 0.0768 | 467 |
| cfDNA | EPCR10 | chr9 | 120950606 | C | T | exonic | CTNNB1 | nonsynonymous SNV | CTNNB1:NM_007614:exon3:c.C98T:p.S33F | 0/1 | 0.016 | 467 |
| cfDNA | EPCR10 | chr9 | 120950609 | G | A | exonic | CTNNB1 | nonsynonymous SNV | CTNNB1:NM_007614:exon3:c.G101A:p.G34E | 0/1 | 0.0105 | 483 |
| cfDNA | EPCR10 | chr9 | 120950630 | C | T | exonic | CTNNB1 | nonsynonymous SNV | CTNNB1:NM_007614:exon3:c.C122T:p.T41I | 0/1 | 0.0659 | 483 |
| cfDNA | EPCR11 | chr6 | 145246771 | C | T | exonic | KRAS | nonsynonymous SNV | KRAS:NM_021284:exon2:c.C35T:p.G12D | 0/1 | 0.504 | 1664 |
| cfDNA | EPCR11 | chr9 | 120950609 | G | A | exonic | CTNNB1 | nonsynonymous SNV | CTNNB1:NM_007614:exon3:c.G101A:p.G34E | 0/1 | 0.117 | 1237 |
| cfDNA | EPCR12 | chr6 | 145246771 | C | T | exonic | KRAS | nonsynonymous SNV | KRAS:NM_021284:exon2:c.C35T:p.G12D | 0/1 | 0.5454 | 1759 |
| cfDNA | EPCR12 | chr9 | 120950609 | G | A | exonic | CTNNB1 | nonsynonymous SNV | CTNNB1:NM_007614:exon3:c.G101A:p.G34E | 0/1 | 0.2737 | 923 |

| | | | | | | | | | | | | |
|---|---|---|---|---|---|---|---|---|---|---|---|---|
| cfDNA | EPCR13 | chr6 | 145246771 | C | T | exonic | KRAS | nonsynonymous SNV | KRAS:NM_021284:exon2:c.C35T:p.G12D | 0/1 | 0.434 | 1440 |
| cfDNA | EPCR13 | chr9 | 120950609 | G | A | exonic | CTNNB1 | nonsynonymous SNV | CTNNB1:NM_007614:exon3:c.G101A:p.G34E | 0/1 | 0.2518 | 1114 |
| cfDNA | EPCR14 | chr9 | 120950630 | C | T | exonic | CTNNB1 | nonsynonymous SNV | CTNNB1:NM_007614:exon3:c.C122T:p.T41I | 0/1 | 0.2071 | 1518 |
| cfDNA | EPCR15 | chr9 | 120950630 | C | T | exonic | CTNNB1 | nonsynonymous SNV | CTNNB1:NM_007614:exon3:c.C122T:p.T41I | 0/1 | 0.2081 | 1428 |
| cfDNA | EPCR16 | chr6 | 145246771 | C | T | exonic | KRAS | nonsynonymous SNV | KRAS:NM_021284:exon2:c.C35T:p.G12D | 0/1 | 0.5972 | 1325 |
| cfDNA | EPCR16 | chr9 | 120950609 | G | A | exonic | CTNNB1 | nonsynonymous SNV | CTNNB1:NM_007614:exon3:c.G101A:p.G34E | 0/1 | 0.087 | 1263 |
| cfDNA | EPCR17 | chr6 | 145246771 | C | T | exonic | KRAS | nonsynonymous SNV | KRAS:NM_021284:exon2:c.C35T:p.G12D | 0/1 | 0.6079 | 2305 |
| cfDNA | EPCR17 | chr9 | 120950609 | G | A | exonic | CTNNB1 | nonsynonymous SNV | CTNNB1:NM_007614:exon3:c.G101A:p.G34E | 0/1 | 0.0659 | 846 |
| cfDNA | EPCR18 | chr6 | 145246771 | C | T | exonic | KRAS | nonsynonymous SNV | KRAS:NM_021284:exon2:c.C35T:p.G12D | 0/1 | 0.2065 | 1025 |
| cfDNA | EPCR18 | chr9 | 120950609 | G | A | exonic | CTNNB1 | nonsynonymous SNV | CTNNB1:NM_007614:exon3:c.G101A:p.G34E | 0/1 | 0.1537 | 1025 |
| cfDNA | EPCR19 | chr6 | 145246771 | C | T | exonic | KRAS | nonsynonymous SNV | KRAS:NM_021284:exon2:c.C35T:p.G12D | 0/1 | 0.1012 | 1711 |
| cfDNA | EPCR19 | chr9 | 120950602 | G | A | exonic | CTNNB1 | nonsynonymous SNV | CTNNB1:NM_007614:exon3:c.G94A:p.D32N | 0/1 | 0.0143 | 940 |
| cfDNA | EPCR19 | chr9 | 120950609 | G | A | exonic | CTNNB1 | nonsynonymous SNV | CTNNB1:NM_007614:exon3:c.G101A:p.G34E | 0/1 | 0.0166 | 1157 |
| cfDNA | EPCR19 | chr9 | 120950618 | C | T | exonic | CTNNB1 | nonsynonymous SNV | CTNNB1:NM_007614:exon3:c.C110T:p.S37F | 0/1 | 0.0137 | 1462 |
| cfDNA | EPCR20 | chr6 | 145246771 | C | T | exonic | KRAS | nonsynonymous SNV | KRAS:NM_021284:exon2:c.C35T:p.G12D | 0/1 | 0.0123 | 1348 |
| cfDNA | EPCR20 | chr9 | 120950602 | G | A | exonic | CTNNB1 | nonsynonymous SNV | CTNNB1:NM_007614:exon3:c.G94A:p.D32N | 0/1 | 0.068 | 1486 |
| cfDNA | EPRC20 | chr9 | 120950606 | C | T | exonic | CTNNB1 | nonsynonymous SNV | CTNNB1:NM_007614:exon3:c.C98T:p.S33F | 0/1 | 0.0211 | 1398 |
| cfDNA | EPCR20 | chr9 | 120950609 | G | A | exonic | CTNNB1 | nonsynonymous SNV | CTNNB1:NM_007614:exon3:c.G101A:p.G34E | 0/1 | 0.0318 | 1142 |
| cfDNA | EPCR21 | chr6 | 145246771 | C | T | exonic | KRAS | nonsynonymous SNV | KRAS:NM_021284:exon2:c.C35T:p.G12D | 0/1 | 0.3549 | 1758 |
| cfDNA | EPCR21 | chr9 | 120950609 | G | A | exonic | CTNNB1 | nonsynonymous SNV | CTNNB1:NM_007614:exon3:c.G101A:p.G34E | 0/1 | 0.0223 | 874 |
| cfDNA | EPCR21 | chr9 | 120950618 | C | T | exonic | CTNNB1 | nonsynonymous SNV | CTNNB1:NM_007614:exon3:c.C110T:p.S37F | 0/1 | 0.014 | 1154 |
| cfDNA | EPCR22 | chr9 | 120950609 | G | A | exonic | CTNNB1 | nonsynonymous SNV | CTNNB1:NM_007614:exon3:c.G101A:p.G34E | 0/1 | 0.034 | 1068 |
| cfDNA | EPCR23 | chr6 | 145246771 | C | T | exonic | KRAS | nonsynonymous SNV | KRAS:NM_021284:exon2:c.C35T:p.G12D | 0/1 | 0.1891 | 909 |
| cfDNA | EPCR23 | chr9 | 120950609 | G | A | exonic | CTNNB1 | nonsynonymous SNV | CTNNB1:NM_007614:exon3:c.G101A:p.G34E | 0/1 | 0.01 | 1461 |
| cfDNA | EPCR24 | chr6 | 145246771 | C | T | exonic | KRAS | nonsynonymous SNV | KRAS:NM_021284:exon2:c.C35T:p.G12D | 0/1 | 0.03 | 1437 |
| cfDNA | EPCR24 | chr9 | 120950609 | G | A | exonic | CTNNB1 | nonsynonymous SNV | CTNNB1:NM_007614:exon3:c.G101A:p.G34E | 0/1 | 0.1104 | 1243 |
| cfDNA | PCR4 | chr6 | 145246771 | C | T | exonic | KRAS | nonsynonymous SNV | KRAS:NM_021284:exon2:c.C35T:p.G12D | 0/1 | 0.0965 | 1518 |

| | | | | | | | | | | | | | |
|---|---|---|---|---|---|---|---|---|---|---|---|---|---|
| cfDNA | PCR4 | chr9 | 120950609 | G | A | exonic | CTNNB1 | nonsynonymous SNV | CTNNB1:NM_007614:exon3:c.G101A:p.G34E | 0/1 | 0.0231 | 893 |
| cfDNA | PCR6 | chr6 | 145246771 | C | T | exonic | KRAS | nonsynonymous SNV | KRAS:NM_021284:exon2:c.C35T:p.G12D | 0/1 | 0.1223 | 1494 |
| cfDNA | PCR6 | chr9 | 120950609 | G | A | exonic | CTNNB1 | nonsynonymous SNV | CTNNB1:NM_007614:exon3:c.G101A:p.G34E | 0/1 | 0.0342 | 1007 |
| cfDNA | PCR7 | chr6 | 145246771 | C | T | exonic | KRAS | nonsynonymous SNV | KRAS:NM_021284:exon2:c.C35T:p.G12D | 0/1 | 0.1314 | 1113 |
| cfDNA | PCR8 | chr6 | 145246771 | C | T | exonic | KRAS | nonsynonymous SNV | KRAS:NM_021284:exon2:c.C35T:p.G12D | 0/1 | 0.0556 | 1830 |
| cfDNA | PCR8 | chr9 | 120950609 | G | A | exonic | CTNNB1 | nonsynonymous SNV | CTNNB1:NM_007614:exon3:c.G101A:p.G34E | 0/1 | 0.0305 | 1089 |
| cfDNA | PCR9 | chr6 | 145246771 | C | T | exonic | KRAS | nonsynonymous SNV | KRAS:NM_021284:exon2:c.C35T:p.G12D | 0/1 | 0.0539 | 924 |
| cfDNA | PCR9 | chr9 | 120950606 | C | T | exonic | CTNNB1 | nonsynonymous SNV | CTNNB1:NM_007614:exon3:c.C98T:p.S33F | 0/1 | 0.0414 | 1099 |
| cfDNA | PCR11 | chr6 | 145246771 | C | T | exonic | KRAS | nonsynonymous SNV | KRAS:NM_021284:exon2:c.C35T:p.G12D | 0/1 | 0.2747 | 118 |
| cfDNA | PCR11 | chr9 | 120950609 | G | A | exonic | CTNNB1 | nonsynonymous SNV | CTNNB1:NM_007614:exon3:c.G101A:p.G34E | 0/1 | 0.028 | 1270 |
| cfDNA | PCR12 | chr6 | 145246771 | C | T | exonic | KRAS | nonsynonymous SNV | KRAS:NM_021284:exon2:c.C35T:p.G12D | 0/1 | 0.0792 | 1428 |
| cfDNA | PCR12 | chr9 | 120950609 | G | A | exonic | CTNNB1 | nonsynonymous SNV | CTNNB1:NM_007614:exon3:c.G101A:p.G34E | 0/1 | 0.0697 | 954 |
| cfDNA | PCR16 | chr9 | 120950609 | G | A | exonic | CTNNB1 | nonsynonymous SNV | CTNNB1:NM_007614:exon3:c.G101A:p.G34E | 0/1 | 0.0438 | 880 |
| cfDNA | PCR17 | chr6 | 145246771 | C | T | exonic | KRAS | nonsynonymous SNV | KRAS:NM_021284:exon2:c.C35T:p.G12D | 0/1 | 0.13 | 1056 |
| cfDNA | PCR17 | chr9 | 120950609 | G | A | exonic | CTNNB1 | nonsynonymous SNV | CTNNB1:NM_007614:exon3:c.G101A:p.G34E | 0/1 | 0.06489 | 1031 |
| cfDNA | PCR18 | chr6 | 145246771 | C | T | exonic | KRAS | nonsynonymous SNV | KRAS:NM_021284:exon2:c.C35T:p.G12D | 0/1 | 0.2334 | 921 |
| cfDNA | PCR18 | chr9 | 120950609 | G | A | exonic | CTNNB1 | nonsynonymous SNV | CTNNB1:NM_007614:exon3:c.G101A:p.G34E | 0/1 | 0.067 | 819 |
| cfDNA | PCR19 | chr6 | 145246771 | C | T | exonic | KRAS | nonsynonymous SNV | KRAS:NM_021284:exon2:c.C35T:p.G12D | 0/1 | 0.0519 | 1592 |
| cfDNA | PCR19 | chr9 | 120950609 | G | A | exonic | CTNNB1 | nonsynonymous SNV | CTNNB1:NM_007614:exon3:c.G101A:p.G34E | 0/1 | 0.0368 | 1024 |
| cfDNA | PCR20 | chr6 | 145246771 | C | T | exonic | KRAS | nonsynonymous SNV | KRAS:NM_021284:exon2:c.C35T:p.G12D | 0/1 | 0.0768 | 985 |
| cfDNA | PCR20 | chr9 | 120950609 | G | A | exonic | CTNNB1 | nonsynonymous SNV | CTNNB1:NM_007614:exon3:c.G101A:p.G34E | 0/1 | 0.0351 | 1132 |
| cfDNA | PCR21 | chr6 | 145246771 | C | T | exonic | KRAS | nonsynonymous SNV | KRAS:NM_021284:exon2:c.C35T:p.G12D | 0/1 | 0.0221 | 1158 |
| cfDNA | PCR21 | chr9 | 120950609 | G | A | exonic | CTNNB1 | nonsynonymous SNV | CTNNB1:NM_007614:exon3:c.G101A:p.G34E | 0/1 | 0.0355 | 1026 |
| cfDNA | PCR21 | chr9 | 120950618 | C | T | exonic | CTNNB1 | nonsynonymous SNV | CTNNB1:NM_007614:exon3:c.C110T:p.S37F | 0/1 | 0.01 | 1398 |
| cfDNA | PCR22 | chr9 | 120950609 | G | A | exonic | CTNNB1 | nonsynonymous SNV | CTNNB1:NM_007614:exon3:c.G101A:p.G34E | 0/1 | 0.029 | 1154 |
| cfDNA | PCR23 | chr6 | 145246771 | C | T | exonic | KRAS | nonsynonymous SNV | KRAS:NM_021284:exon2:c.C35T:p.G12D | 0/1 | 0.0217 | 1051 |
| cfDNA | PCR23 | chr9 | 120950609 | G | A | exonic | CTNNB1 | nonsynonymous SNV | CTNNB1:NM_007614:exon3:c.G101A:p.G34E | 0/1 | 0.028 | 1552 |

| | | | | | | | | | | | | | |
|---|---|---|---|---|---|---|---|---|---|---|---|---|---|
| cfDNA | PCR24 | chr9 | 120950609 | G | A | exonic | CTNNB1 | nonsynonymous SNV | CTNNB1:NM_007614:exon3:c.G101A:p.G34E | 0/1 | 6.48E-02 | 873 |

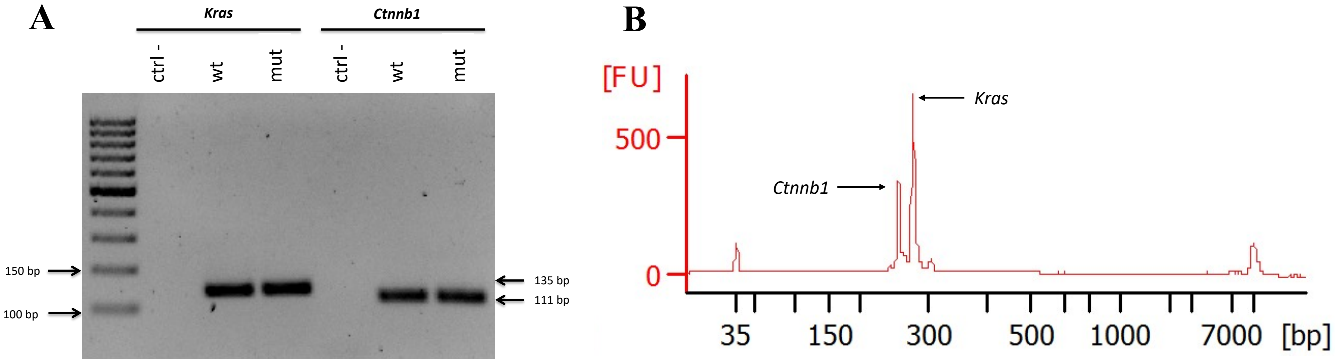


**Supp. Figure 1.** A) Representative image of the size of the PCR products for *Kras* and *Ctnnb1* used for library preparation. B) Final library of *Kras* and *Ctnnb1* evaluated in bioanalyzer. Barcodes were added to the PCR products at 5' and 3', increasing their size by 120 base pairs.

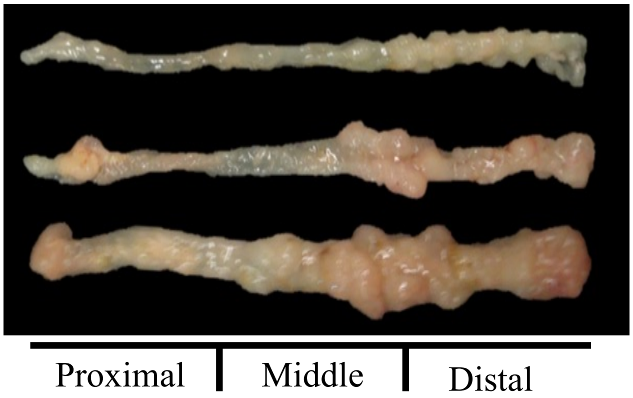


**Supp. Figure 2.** Representative image of CRC development in the AOM/DSS model taken at the end of the experiment. The tumors were developed in the middle and distal part of the colon.

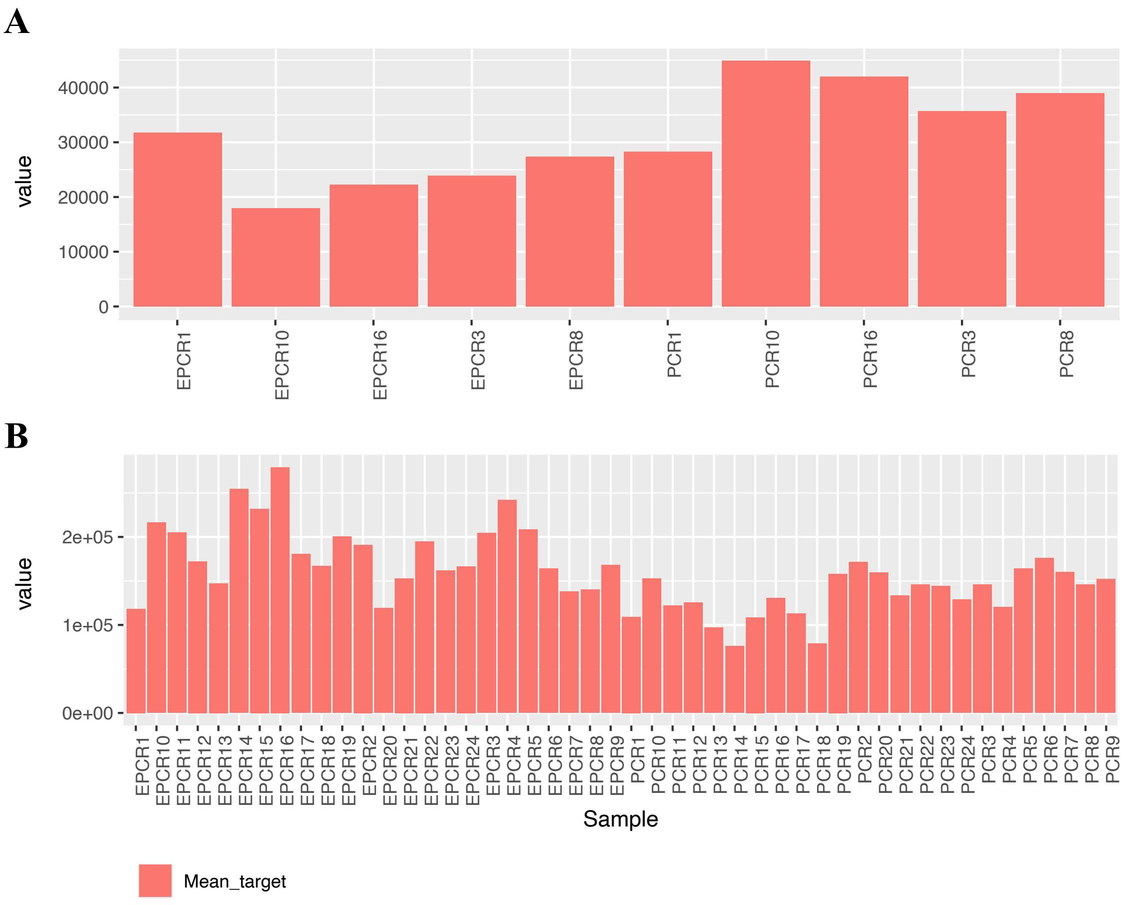


**Supp. Figure 3.** Depth of sequencing for all the samples. A) Sequencing depth in tumor samples (mean 31,310.68 ±8,924.165). B) sequencing depth in cfDNA (mean 159,509.8 ±43,030.71).

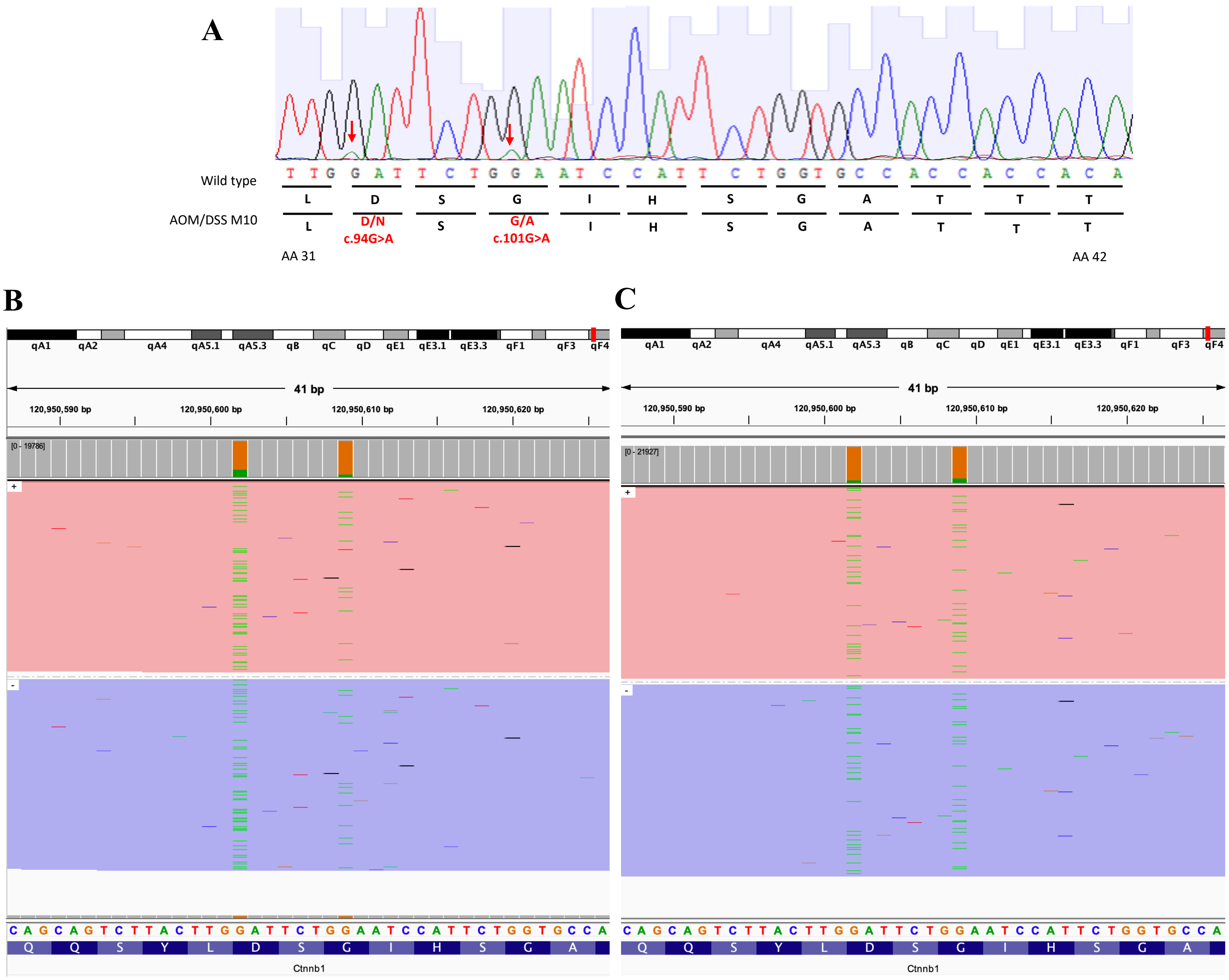


**Supp. Figure 4.** Representative images of the mutational profiling of the tumor of mouse M10. A) Sanger sequencing electropherogram showing a mutation in the *Ctnnb1* gene. B) Local mutation profile of the *Ctnnb1* gene in the Integrative Genomic Viewer after E-ice-COLD-PCR amplification and massive parallel sequencing. C) Local mutation profile of the *Cttnnb1* gene in the Integrative Genomic Viewer after PCR and massive parallel sequencing.
